# The Biocontrol and Growth-Promoting Potential of *Penicillium* spp. and *Trichoderma* spp. in Sustainable Agriculture

**DOI:** 10.3390/plants14132007

**Published:** 2025-06-30

**Authors:** Wenli Sun, Mohamad Hesam Shahrajabian, Lijie Guan

**Affiliations:** 1 National Key Laboratory of Agricultural Microbiology, Biotechnology Research Institute, Chinese Academy of Agricultural Sciences, Beijing 100086, China; hesamshahrajabian@gmail.com; 2 College of Environmental and Safety Engineering, Shenyang University of Chemical Technology, Shenyang 110142, China; guanlijie6868@syuct.edu.cn

**Keywords:** fungal endophytes, plant-growth-promoting fungi, phytohormones, sustainability

## Abstract

Plant-growth-promoting fungi (PGPF) play a central role in promoting sustainable agriculture by improving plant growth and resilience. The aim of this literature review is to survey the impacts of *Trichoderma* spp. and *Penicillium* spp. on various agricultural and horticultural plants. The information provided in this manuscript was obtained from randomized control experiments, review articles, and analytical studies and observations gathered from numerous literature sources such as Scopus, Google Scholar, PubMed, and Science Direct. The keywords used were the common and Latin names of various agricultural and horticultural species, fungal endophytes, plant-growth-promoting fungi, *Trichoderma*, *Penicillium*, microbial biostimulants, and biotic and abiotic stresses. Endophytic fungi refer to fungi that live in plant tissues throughout part of or the entire life cycle by starting a mutually beneficial symbiotic relationship with its host without any negative effects. They are also capable of producing compounds and a variety of bioactive components such as terpenoids, steroids, flavonoids, alkaloids, and phenolic components. *Penicillium* is extensively known for its production of secondary metabolites, its impact as a bioinoculant to help with crop productivity, and its effectiveness in sustainable crop production. The plant-growth-promotion effects of *Trichoderma* spp. are related to better absorption of mineral nutrients, enhanced morphological growth, better reproductive potential and yield, and better induction of disease resistance. Both *Penicillium* spp. and *Trichoderma* spp. are effective, affordable, safe, and eco-friendly biocontrol agents for various plant species, and they can be considered economically important microorganisms for both agricultural and horticultural sciences. The present review article aims to present the most up-to-date results and findings regarding the practical applications of two important types of PGPF, namely *Penicillium* spp., and *Trichoderma* spp., in agricultural and horticultural species, considering the mechanisms of actions of these species of fungi.

## 1. Introduction

Plant-growth-promoting microorganisms (PGPM) include fungi, bacteria, and other microorganisms that improve the performance of plants as well as increase their nutrient absorption under both abiotic and biotic stress conditions, with notable potential advantages for the growth and development of seedlings [1,2,3]. Plant-growth-promoting fungi (PGPF) are a group of non-pathogenic soil-borne filamentous fungi that exert positive effects on plants; these can enhance plant growth both indirectly and directly by producing phytohormones, fixing nitrogen, and inducing systemic resistance [4,5,6]. *Trichoderma* and *Penicillium* are the most effectual organisms among the numerous phosphate-solubilizing fungi that can dissolve insoluble phosphate and improve its uptake by plant roots, which are the main reasons for the plant’s appropriate growth and development [7,8,9]. In many research studies, it has been reported that a combination of biologically active compounds of plant or microbial origin, or their synthesized analogues, together with fungicides is a promising and sustainable approach to controlling crop diseases [10]. Fungi show significant metabolic properties because of their sophisticated genomic network, and they have notable importance because of their different roles in our world, such as in their applications in agriculture, industry, and medicine. Filamentous fungi of the genera *Trichoderma* have been widely studied because they can interact with and colonize plant roots via different processes that can increase plant growth through phytohormone synthesis, nutrient absorption, tolerance to abiotic stress, and the induction of systemic resistance as well as by acting as biological control factors. Their applications and their wonderful roles in environmentally friendly agricultural practices for different crops such as lettuce, soybean, wheat, corn, tomato, beans, etc., have been mentioned in previous research studies [11,12,13,14].

Some species of *Penicillium* produce different biologically active compounds [15,16,17], some of them with an extensive range of fungicidal action [18,19] and plant growth-stimulating activity [20]. Different species of *Penicillium* can significantly improve the dry and fresh weight of shoots as well as enhance the chlorophyll content [21]; for example, *P. pinophilum* can form arbuscular mycorrhizae, which can enhance the nitrogen content, photosynthesis rate, P content, and plant dry weight of strawberry plants [22]. The free-living soil fungi *Trichoderma* spp. are potential biological control agents of plant-parasitic nematodes [23], and they can control a wide range of economically notable plant pathogenic fungi, nematodes, bacteria, and viruses [24,25]. *Trichoderma* is the asexual stage of the filamentous Hypocrea genus belonging to the Ascomycota fungi division [26], and it is considered the most frequently isolated soil microorganism [27]. As PGPF, *Penicillium* spp. are known to grown in extreme conditions such as areas with high salt concentrations, and their application has increased in recent years due to their noticeable ability to increase salt-stress tolerance in different plant species as well as increase the levels of chemical components and improve the antioxidative system through the production of organic acids. *Penicillium* species can inhibit pathogens in the soil, and they are also highly effective in fixing potassium, dissolving phosphorus, and solubilizing soil-bound phosphate. In fact, *Penicillium* species can increase the absorption of phosphorus, improve soil conditions, help plants defend against diseases, and improve seed protection in plants facing pathogens. There has not been enough research on the effects of *Penicillium* fungi on plants, and more research is needed to study the effects of *Penicillium* fungi on soil phosphorus cycling and phosphorus uptake in plants as well as their importance in increasing plant growth. *P. citrinum* has been found to be a common endophytic fungus in cereal plants and has been isolated from various environmental conditions, ranging from permafrost sediments to agricultural fields and forest soils [28].

*Trichoderma* species such as *T. harzianum*, *T. viride*, *T. atrovitide*, *T. hamatum*, *T. virens*, and *T. longibrachiatum* are common in root and soil ecosystems, where they can establish root colonization, enhance crop productivity, growth and development, increase the use and uptake of nutrients, and improve resistance to abiotic stresses [29,30,31]. Chinnaperumal et al. [32] reported that *Trichoderma viride* can be considered an important inhibitor of the development of *Helicoverpa armigera*, with no negative impacts on earthworms. Panchalingam et al. [33] showed the positive effects of the combined use of a *Streptomycetes* consortium and *Trichoderma* as a potential biocontrol agent against the brown root rot pathogen *Pyrrhoderma noxium* (Corner) L.W. Zhou and Y.C. Dai in soil. The volatile organic compounds (VOCs) emitted by *Trichoderma* spp. have various effects against plant pathogenic fungi such as *Sclerotinia nivalis*, *Cylindrocarpon destructans*, *Botrytis cinerea*, *Alternaria panax*, *Stagonosporopsis cucurbitacearum*, *Penicillium oxalicum*, *Ganoderma* sp., *Fusarium oxysporum*, *Sclerotium rolfsii*, and *Sclerotinia sclerotiorum* [34,35]. *Trichoderma* is highly known as a biostimulant because of its main effects on plants, such as stimulating higher nutrient uptake efficiency, improving the rate of photosynthesis and metabolism, and as a potential biocontrol agent. Its different types of products can increase productivity and plant growth, and it can be applied as a good biocontrol agent that does not negatively influence other microorganisms in the soil. *Trichoderma*-based products have been identified as appropriate biological control agents for different plant pathogens, and they increase resistance to biotic stresses; thus, its different species have gained importance as microbial plant biostimulants in both agricultural and horticultural sciences. They can also mitigate the detrimental effects of abiotic stresses, improve nutritional quality and yield, and enhance plant growth. As important endophytic fungi, *Trichoderma* spp. can interact with other strains of the microbial community in the rhizosphere, which shows the importance of evaluating the ecological effects of different soil management and microbial-based biostimulants on the soil ecosystem.

Endophytes are known as microorganisms that can be found inside plant tissue without triggering any adverse impacts; rather, they reveal a positive influence on crop yield and plant growth, showing a high capacity for decreasing the need for fertilizer and stimulating plant biochemical components by providing osmoregulation, antioxidative defenses, and affecting nutrient uptake effectiveness during various biotic and abiotic stresses [36,37,38,39,40]. Different endophytic bacteria and fungi, including *Bacillus*, *Actinomycetes*, *Trichoderma*, *Pseudomonas*, and *Epicoccum*, were reported to elicit plant disease tolerance in cacao, potato, chili, cotton, and tomato [41]. It is also very important to choose suitable fungal strains, as some strains of the genera *Penicillium* are pathogens of crops and animals; *P. allii* is a pathogen of garlic, *Penicillium* spp. is a pathogen of pear, apple, and citrus fruit [42,43], and *P. glabrum* is a pathogen of garlic and onion [44].

**Figure 1:**
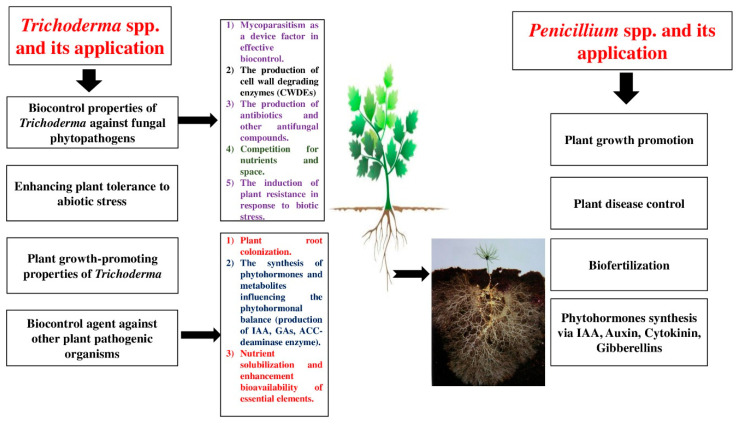
Studying the advantages of *Penicillium* spp. and *Trichoderma* spp.

Plant–fungal interactions can be categorized as mutualistic, commensalistic, and pathogenic [45]. The most important mechanisms of PGPF involve the production of plant growth regulators such as abscisic acid, ethylene, gibberellins, cytokinins, and auxins; the production of organic acids and siderophores such as iron, zinc, potassium, phosphorus, and nitrogen; an increase in water uptake; the production of hydrolytic enzymes such as cellulases, pectinases, laccases, and xylanases; reductions in the amount of ethylene; relief from various abiotic stresses in harsh environments; and the induction of plant defense mechanisms against pathogens [46,47]. *Trichoderma* spp. and *Penicillium* spp. can be further studied to improve their ability as effective biocontrol agents; moreover, they show various antagonistic mechanisms against plant pathogens such as mycoparasitism, antibiosis, the promotion of plant growth, and competition for nutrients and space. They can be also considered important candidates for application in green technologies because of their great biostimulatory and biofertilization potential. The aim of this literature review is to survey the impacts of *Trichoderma* spp. and *Penicillium* spp. on various agricultural and horticultural plants, with the information provided in this manuscript obtained from randomized control experiments, review articles, and analytical studies and observations gathered from numerous literature sources such as Scopus, Google Scholar, PubMed, and Science Direct. The keywords used were the common and Latin names of various agricultural and horticultural species, fungal endophytes, plant-growth-promoting fungi, *Trichoderma*, *Penicillium*, microbial biostimulants, and biotic and abiotic stresses. The benefits and advantages of applications of both *Penicillium* spp. and *Trichoderma* spp. are presented in Figure 1.

## 2. *Penicillium* spp. as Plant-Growth-Promoting Fungi with Biocontrol Properties

Beneficial *Penicillium* spp. are used in the production of antibiotics, with the most famous advantage being the production of penicillin by *P. chrysogenum*, while some species produce griseofulvin, which is an antifungal drug. Other species are used in food and beverage production; in bioremediation, as some *Penicillium* species can break down different contaminants and pollutants in water and soil; and some species produce enzymes used in different industrial processes. They are also used to promote plant growth and stress tolerance, as certain species can help plants tolerate environmental stresses like drought, salinity, and heavy metals, and they can also increase the nutrient availability and promote root development in various plants. It is also reported that some species such as *P. roqueforti* and *P. camemberti* are important for cheese production. When using *Penicillium* for specific applications, it is essential to select the appropriate strain due to variations in their capabilities and properties.

*Pencillium* spp. are known to grow in extreme environments such as those with high salt concentrations, and some strains and species have been reported as PGPF with the ability to increase salt tolerance in different plant species [48,49,50,51,52]. *Penicillium* spp. colonize plant roots via several mechanisms, including endophytism, rhizosphere association, and mycorrhizal-like interactions [53,54,55]. They are often involved in a range of complex interactions with plants and use various strategies and develop distinct ways to mediate improvements in seed vigor, seed germination, plant growth, flowering, and productivity of host plants.

Root colonization by *Penicillium* spp. or its cell-free filtrate elicited an induced systemic resistance against infection by *Pseudomonas syringae* pv. Tomato DC3000 (Pst), leading to restricted pathogen growth and disease development [56]. Hossain et al. [56] showed that signal transduction leading to a GP16-2-mediated induced systemic resistance (ISR) requires responsiveness to JA and ET in an NPR1-dependent manner, while the cell-free filtrate (CF)-mediated ISR shows that salicylic acid (SA)-, JA-, ET-, and NPR1-dependent signaling is dispensable (at least individually). Moreover, root colonization by GP16-2 is not connected with a direct effect on the expression of known defense-related genes; however, it potentiates the activation of JA/ET-inducible basic chitinase (ChitB), which only becomes apparent after infection by Pst. But CF-mediated ISR is partly associated with the direct activation of marker genes responsive to both SA and JA/ET signaling pathways and partly associated with priming, leading to the activation of JA/ET-inducible ChitB and hevein-like protein (Hel) genes.

Species of *Penicillium* are ubiquitous fungi because of their ability to grow over a wide range of environments and conditions and their undemanding nutritional requirements; moreover, the genus is one of the largest groups of fungi. Its important species are *P. chrysogenum*, *P. citreonigrum*, *P. citrinum*, *P. digitatum*, and *P. janthinellum* [57,58,59]. Endophytic *Penicillium* species have different applications in (1) biotechnology—such as in the production of enzymes (such as lipase, inulinase, amylase, protease, cellulase, xylanase, β-glucosidase, etc. [60]), biotransformation [61,62], and the synthesis of nanoparticles like silver nanoparticles [63,64]; (2) agriculture—such as in phytoremediation, biocontrol, and insecticidal activities [65,66,67]; for example *P. chrysogenum* QEN-24S displayed potent activity against the pathogen *Alternaria brassicae*, which infects important crops such as oil seed rape, cabbage, and broccoli [67], while penicisteroids A and B, two new polyoxygenated steroids obtained from the culture extract of *P. chrysogenum* QEN-24S, revealed potent antifungal and cytotoxic activity in preliminary bioassays [65,66]; and (3) drug discovery—such as in immuno-suppressive [67], antifibrotic [68,69], neuroprotective [70], antidiabetic [70], anti-obesity [70,71,72], anti-inflammatory [22], antioxidative [22], anticancer, antiparasitic [73], antiviral [73,74], and antimicrobial [74] applications.

The species are also well-known for their unique potency in removing pollutants like heavy metals, such as mercury, lead, chromium, arsenic, and cadmium, from various ecosystems [75,76,77,78,79]. Sonderegger et al. [79] found that small, cysteine-rich, and cationic antifungal proteins (Aps) from filamentous ascomycetes, such as NFAP from Neosartorya fischeri and PAF from *P. chrysogenum*, are promising candidates for novel drug development. Garcia-Estrada et al. [80] showed that penicillin biosynthesis by *P. chrysogenum* is one of the best-characterized biological process from molecular, genetic, biochemical, and subcellular points of view, and omics studies have been conducted on this filamentous fungus over the last decade, which have contributed to gathering a deep knowledge about the molecular mechanisms underlying the improved productivity in industrial strains.

It has been reported that a halotolerant phenylacetate-degrading fungus *P. chrysogenum* CLONA2 strain can produce non-aromatic natural penicillin rather than benzylpenicillin, and it can be an appropriate option for aromatic compounds remediation in high-salinity regions [74]. *Penicillium* CLONA2, isolated from a salt mine at Algarve (Portugal), was identified as a variant of *P. chrysogenum* using ITS-5,85 rDNA and the D1/D2 domain of 28S rDNA sequences. Due to the ability of *P. chrysogenum* CLONA2 to degrade aromatic compounds, this strain can be considered an important organism for aromatic compound remediation in high-salinity environments. Garcia-Rico et al. [81] found that the heterotrimeric Gα protein, Pga1, of *P. chrysogenum* controls conidiation, vegetative growth, and secondary metabolite production. The secondary metabolites of *P. chrysogenum* are chrysogine, penicillins, sorrentanone, secalonic acids, and PR-toxins [82,83,84]. Guijarro et al. [85] reported that *P. frequentans* (Pf909) could decrease brown rot caused by *Monilinia* spp. in stone fruit, and that it could survive and establish actively in a broad range of climatic conditions. Arunthirumeni et al. [86] concluded that *Penicillium* spp. can produce secondary components that are effectual for the control of Spodoptera litura and Culex quinquefasciatus larvae. The combined application of a commercial azoxystrobin-based fungicide and the *P. chrysogenum* F-24-28 strain (DMP) induced to prolonged growth inhibition of *F. culmorum*, *F. graminearum*, and *F. oxysporum* at fungicide concentrations, which suggests that this approach can be used to control crop diseases [87]. Sikandar et al. [88] reported that *P. chrysogenum* Snef1216 has the potential to be used against Meloidogyne incognita, the main root-knot nematode, which is one of the most dangerous nematodes due to its high reproduction rate and extensive host range. *P. citrinum* has been found to be a common positive endophytic fungus of cereal plants like soybean and wheat [89]. Nguyen et al. [90] reported that *P. citrinum* can be used for the biological control of *Plutella xylostella* and *Spodoptera litura* in some crops. The non-volatile components produced by *P. simplicissimum* CEF-818, which is an endophyte from *Gossypium hirsutum*, were shown to strongly suppress the growth of the plant pathogen *Verticillium dahliae* isolate Vd080 [91,92,93]. It was shown that *P. commune* MC-9L could act as a biological agent against *Sclerotinia* sp. in fumigation assays [94,95]. It was also found that *P. crustosum* and *P. chrysogenum* isolated from *Teucrium polium* produced ammonia and IAA and showed a high phosphate solubilization capacity [96].

*P. janthinellum* can be used for the biological control of phytophthora root rot in azalea [97], while *P. citrinum* can noticeably enhance chemical metabolite production and yield [98]. Dry mycelium of *P. chrysogenum* is important in controlling fungal diseases in cotton [99], and it is also effective in increasing the germination index, germination rate, and seed germination rate in cucumber [100]. Moreover, *P. chrysogenum* can improve the flowering activity of jujube [101] and improve the yield and yield components of maize [102] and pearl millet [103]. Dry mycelium of *P. chrysogenum* can increase the resistance of seedlings against the root-knot nematode *Meloidogyne javanica* in tomato [104], and *P. janthinellum* LK5 can increase the resistance of plants against salinity stress [105] and increase metal phytoextraction while promoting crop physiological homeostasis [106]. The impacts of various species of *Penicillium* spp. on the yield and yield components of various plants are shown in Table 1.

## 3. *Trichoderma* spp. as Plant Growth-Promoting Fungi with Biocontrol Properties

The species of fungi belonging to *Trichoderma* genus are endophytic saprophytes, which can easily colonize the host root surface of plants, induce resistance, promote plant health as a biocontrol agent, and increase plant growth [125]. *Trichoderma* is highly known as a biostimulant because of its main impacts on plants, such as stimulating a higher nutrient uptake efficiency, improving the rate of metabolism and photosynthesis, and as a potential biocontrol agent. Its different types of products can increase productivity and the plant growth, and it can be applied as a good biocontrol agent without negatively influencing other microorganisms in the soil. The genus *Trichoderma* possesses mycoparasitic potential against pathogenic fungi [126]. It also has high potential for degrading pollutants [127,128]. At first, *Trichoderma* colonizes between living plant cells, resembling the early stages in an attack by soil-borne pathogens, and to start colonization, conidial germ tubes or hyphae growing toward and near the root in the rhizosphere almost adhere to the root epidermis [129,130].

**Figure 2:**
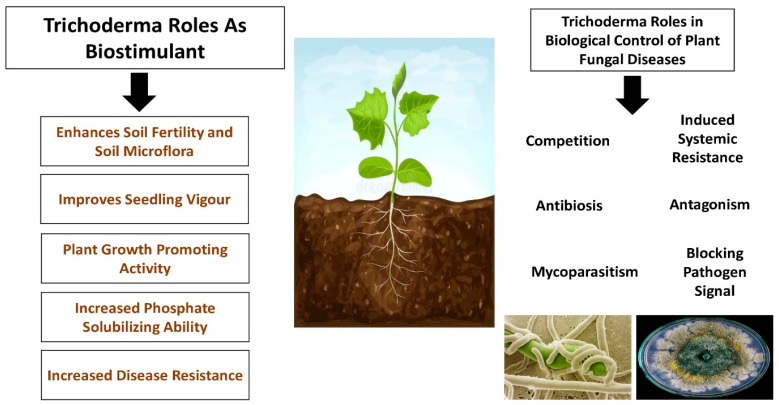
Plant biostimulatory actions of *Trichoderma* spp.

However, Pfordt et al. [131] reported that *T. afroharzianum* has been found as a pathogen causing ear rot disease on maize in Italy, France, and Germany, leading to massive infections on maize cobs, which has been confirmed even in other studies [132,133]. In 2020, *T. afroharzianum* was reported for the first time in Europe as an ear rot disease in maize [132]. The *T. afroharzianum* ear rot was characterized by a massive production of gray–green spores in the interkernel regions and on the outer surface of the husks, causing a significant reduction in the dry matter content and the premature germination of kernels [132,133]. The most important activities of *Trichoderma* spp. are shown in Figure 2.

## 4. Journey of *Trichoderma* spp. Species

In the year 1794, the name *Trichoderma* was introduced, and in 1865, the sexual stage of *T. viride* (*Hypocrea rufa*) was reported. In 1932, the first evidence that *T. lignorum* (*Hypocrea virens*) has mycoparasitic and biocontrol abilities was presented, and in 1934, the first anti-microbial compound from *Trichoderma* spp., namely gliotoxin, was discovered. In 1957, the discovery of the effect of light on *T. viride* was reported. In general, when in darkness, *Trichoderma* grows indefinitely as mycelium, and a brief pulse of light applied to the actively growing zone of the mycelium leads to the formation of dark green mature conidia, forming a ring at what was the edge of the colony when light was applied; the first event induced by light is a fast, first-order, photochemical reaction that does not need the presence of molecular oxygen and is independent of temperature [134]. In 1972, the demonstration of biocontrol activity of *T. harzianum* against *Sclerotium rolfsii* in field conditions was reported, and in 1983, the cloning of the first *Trichoderma* species, which was *T. reesei*, was performed. In 1986, the report on the expression of growth promotion in the root was published, while in 1987, the successful transformation of *T. reesei* was reported, and in 1989, the first registration of a commercial formulation was reported. Evidence of the cloning of lectin-coated fibers by *Trichoderma* species was presented in 1992, and in 1998 and 1999, the identification of factors that induced the genes for mycoparasitism and the demonstration of *Trichoderma* internal colonization of plant roots was accomplished. In 2011, a genome comparison of three species of *Trichoderma* was conducted [135], and in 2022, five new *Trichoderma* species were reported [136,137,138,139].

## 5. *Trichoderma* spp. and Prospects for Application in Agriculture, Horticulture, and Organic Farming

Some scientific publications have shown that *Trichoderma* species can also significantly influence plant phytohormone networking and production by the secretion of enzymes that can change plant ethylene levels [140].

*T. viride* and *T. longibrachiatum* have shown effective larvicide impacts against dengue mosquitoes such as *Aedes albopictus* and *Aedes aegypti* [141]. *T. asperellum* also possesses both plant-growth-promoting and biocontrol activities [142]. It has also been reported that the combined application of *T. koningii* and *T. virens* with extracts of *Chlorella vulgaris* are significantly effective against severe disease-late wilt (LWD) of maize incited by *Cephalosporium maydis* Samra under both field and greenhouse conditions [143]. Previous studies confirmed the IAA production potential of *Trichoderma* species, including *T. harzianum*, *T. asperellum*, *T. longibrachiatum*, *T. pinnatum*, *T. virens*, *T. asperelloides*, *T. guizhouense*, *T. atrobrunneum*, *T. simmonsii*, and *T. paratroviride* [144]. It has been reported that one of the major and effective ways to control phytopathogenic microorganisms and to reduce the adverse impacts of heavy metals on plants is via the application of *T. harzianum* [145]. The presence of alfalfa seedlings with *Trichoderma* increased the available nutrient (K, P, and N) content in the soil and the alfalfa biomass [146], as *Trichoderma* acts as a nutrient mobilizer, improving the yield, yield components, and quality traits of the crops [147]. *Trichoderma* spp. have been found to degrade chlorpyrifos, benzimidazole fungicide, 2,2-dichlorovinyl dimethyl phosphate, and penthiopyrad [148]. *T. hamatum* can be considered for its plant-growth promotion [149] and various biocontrol activities, and it also possesses different beneficial activities such as antioxidative activity [150], antimicrobial properties [151], herbicidal activity [152], and insecticidal activity [153,154].

*Trichoderma* spp. has shown biocontrol activity by producing certain hydrolytic and antibiotic enzymes, such as β-1,3-glucanase and chitinase, which facilitate cell-wall degeneration and, ultimately, cause the death of pathogenic microorganisms [155]. Transcriptional parameters such as MYBs, WRKYs, and MYCs have shown important functions in priming as they act as regulatory nodes in the transcriptional network of systemic defense after stress recognition, and when it comes to long-lasting priming, *Trichoderma* spp. may have roles in the plants’ epigenetic regulation via DNA (hypo)methylation, histone replacement and modification, RNA-directed DNA methylation (RdDM), and DNA (hypo)methylation, and the inheritance of epigenetic markers can improve growth promotion and enhance the resistance of plants [156,157]. The most effectual biocontrol characteristics are pertain to *T. harzianum*, *T. virens*, *T. pseudokoningii*, *T. koningii*, *T. asperellum*, *T. longibrachiatum*, *T. viride*, and *T. polysporum*, which have a considerable effect on the development of plant diseases caused by *F. culmorum*, *F. oxysporum*, *Verticillium dahliae*, *Gaeumannomyces graminis* var. *tritici*, *Pythium aphanidermatum*, *Sclerotium rolfsii*, and *R. solani* in both field and greenhouse conditions [158].

Some researchers have reported that since many *Trichoderma* species are fungal parasitoids and symbiotic, they need to produce secondary metabolites and degradation enzymes to obtain nutrients from the host; thus, they have been developed as biocontrol factors for plant diseases [159]. *T. hamatum* has shown antibacterial effects on *Xanthomonas campestris* pv. *armoraciae*, and *Xanthomonas euvesicatoria* [160,161], *Bacillus subtilis*, *Staphylococcus aureus*, *Pseudomonas aeruginosa*, *Serratia*, and *Acidovorax avenae* [162], and *Ralstonia solanecearum* [160,163]. *T. hamatum* has also shown antifungal activity against *Rhizoctonia solani* in radish [164], *Sclerotinia sclerotiorum* in lettuce [164], *Magnaporthe oryzae* in *Arabidopsis thaliana* [165,166,167,168], *Sclerotinia asari* in *Asarum rhizosphere* [169], *F. proliferatum*, *F. solani*, and *F. oxysporum* in *Aconitum carmichaelii* Debx [170], *Lasiodiplodia theobromae* in *Macadamia integrifolia* [170], and *Sclerotinia sclerotiorum* in *Arabidopsis thaliana* [171]. Different species of *Trichoderma*, like. *T. asperellum*, *T. atroviride*, *T. harzianum*, *T. viride*, *T. citrinoviride*, *T. koningii*, and *T. hamatum*, can produce 6-pentyl-alpha-pyrone (6PP), which is a lactone with a coconut-like aroma that has special potency for increasing root hair development and root branching [172,173] and for improving plant health and growth [174]. The occurrence of *Trichoderma* spp. species in different environments is shown in Table 2.

Compounds synthesized by *Trichoderma* spp. that are involved in plant interactions are IAA in *T. virens*, GA_3_ in *Trichoderma* spp., ABA in *T. virens* and *T. atroviride*, ethylene in *T. atroviride*, jasmonic acid (JA) in *T. asperellum*, and salicylic acid (SA) in *T. atroviride* [185]. A novel type II hydrophobin secreted by the biocontrol strain MK1 of *T. longibrachiatum* was characterized and isolated, and the corresponding gene (Hytlo1) was shown to have multiple functions in the *Trichoderma*–plant pathogen three-way interactions, while the purified protein showed direct antifungal and plant-growth promotion (PGP) activities as well as microbe-associated molecular patterns [185]. In this experiment, leaf infiltration with hydrophobin systemically enhanced the resistance to pathogens and activated defense-related responses involving phytoalexin, oxylipin, superoxide dismutase, reactive oxygen species, and pathogenesis-related formation or activity; moreover, hydrophobin stimulated root formation and growth, and a targeted knock-out of Hytlo1 significantly decreased both the antagonistic and PGP effects of the wild-type strain [185]. Polyketides in *T. virens* and *Trichoderma* sp. SCSIO41004; terpenes in *T. virens*, *T. harzianum* P1-4, *T. citrinoviride*, and *T. harzianum* R5; VOCs in *T. atroviride* and *T. arundinaceum*; and hydrophobin in *T. virens*, *T. atroviride*, and *T. asperellum* have different functions, such as in plant-growth promotion, and facilitate the plant–microbe interactions in the rhizosphere [186,187]. Claudia et al. [186] showed that the product of the TvCyt2 gene from *T. virens* encoded a new protein homologous to cytochrome p450, which is down-regulated at the beginning of the *Trichoderma*–*Arabidopsis* interaction, and Arabidopsis plants co-cultivated with the *OETvCyt2* strains showed a stronger induction of systemic acquired resistance than plants co-cultivated with the WT strain, as well as increases in biomass and fitness, which show that the *TvCyt2* gene is involved in secondary metabolite biosynthesis, and this can increase the antagonistic activity toward phytopathogenic fungi and the capacity to promote plant growth [186].

Some of the species of *Trichoderma* are active in regulating different genes in plants, such as *T. asperelloides*, which can up-regulate the MDAR gene in *Arabidopsis* and cucumber, which can increase osmo-protection and oxidative stress [188]; *T. atroviride* and *T. virens*, which can up-regulate AtERD14 in *Arabidopsis* and mitigate cold stress effects; *T. parareesei*, which can up-regulate PYL4, ERF1, ACCO1, and NCED3 in rapeseed, thus improving tolerance to salinity and drought; *T. longibrachiatum* in wheat, which can up-regulate CAT, POD, and SOD and increase the resistant of plants to salinity [189]; *T. harzianum* in tomato, which can improve the tolerance of seedlings to cold via P5CS and TAS14 [190]; *T. harzianum*, which regulates GST1 and Lox in potato, which can improve plant resistance to diseases [191]; and *T. asperellum*, which can up-regulate PdPapARF1 in poplar, which can promote growth and defense responses [192].

Some parameters that may encourage the market for *Trichoderma*-based biofungicides are that they should be broad-spectrum in action, show consistent field performance, have an extensive lifespan, be a cost-effective product, have easy accessibility and improved delivery systems, and there should be social awareness of their benefits among farmers [193]. *Trichoderma* spp. as potential biocontrol agents (BCAs) can be used for the effective management of different soil, foliar, and post-harvest plant pathogens, and they have gained more attention in recent years.

Some of the identified genes from *Trichoderma* species have different roles during the biocontrol interaction with phytopathogens, such as Tvsp1 from *T. virens*, which can protect cotton seedlings against *R. solani* [194]; Tag3 from *T. asperellum*, which is responsible for glucanase production for cell-wall degradation [195]; TgaA and TgaB from *T. virens*, which has shown biocontrol effectiveness for the management of *R. solani* and *Sclerotium rolfsii* [196]; ThPG1 from *T. harzianum*, which is needed for beneficial interactions between *T. harzianum* and the host [197]; ThPRT2 from *T. harzianum*, which has shown mycoparasitism activity against *Botrytis cinerea* [198]; tri5 of *T. brevicompactum* IBT40841, which has antifungal activity and has been used in the production of trichodermin against fungi causing infections in the human body [199], TvGST of *T. virens*, which provides enhanced tolerance against cadmium stress [200]; TrCCD1 of *T. reesei*, which can facilitate pigment production and hyphal growth [201]; egl1 of *T. longibrachiatum*, which shows antagonistic activity against *Pythium ultimum* [202]; qid74 of *T. harzianum*, which has roles in plant biofertilization and the root architecture [203]; tac1 of *T. virens* IMI 304061, which shows mycoparasitism against *S. rolfsii* and *R. solani* [204]; TrCCD1 of *T. reesei*, which can promote conidia formation and elongation of fungal hyphae; XI 1 of *Trichoderma* strain Y, which is helpful in hemicellulose breakdown [205]; tvhydii1 of *T. reesei*, which is important in mycoparasitism and plant–fungus interactions [206], gpr1 of *T. atroviride*, which is needed for the stability of cell walls and hyphal growth [207]; ipa-1 of *T. virens*, which has a role in antibiosis against *R. solani* [208]; TasXyn24.2 and TasXyn29.4 of *T. asperellum*, which can induce resistance and increase growth in seedlings [208], and agl1 of *T. atroviride*, which can be used for the biological control of plant pathogens. It has been reported that the application of grains (200 g + sugar (1%) + *T. harzianum*) showed 12.96% effectiveness in the management of chili wilt disease [209], and the application of vermicompost fortified with *Trichoderma* induced a reduction of 10.01% in the incidence of wilt in chili [209]. A mixture of ground grain + sugar solution (1%) + *T. harzianum* and the combined application of decomposed cow dung + a *Trichoderma* formulation has positive impacts on maize seeds [210], while the application of partially crushed grain + sugar (1%) solution + distilled water and the combined application of beech, fir, and chestnut + conidial suspensions of *T. atroviride* + distilled water + soy flour could increase the yield [211].

Guzman-Guzman et al. [212] reported that the biocontrol mechanisms of *Trichoderma* spp. against potential pathogens include antibiosis, parasitism, secondary metabolite production, plant defense system induction, and competition. Different *Trichoderma* species used in agriculture have different biocontrol characteristics; for example, *T. atroviride*, *T. viride*, *T. longibrachiatum*, *T. virens*, *T. asperellum*, and *T. harzianum* have demonstated parasitism, competition, plant defense induction, priming, secondary metabolite production, and antibiosis [213,214]. Chan et al. [215] reported that the *T. harzianum* strain CE92 can be used as a potential biocontrol agent for pathogenic wood rot fungal species such as *Rigidoporus microporus*, *Phellinus noxius*, and *Fulvifomes siamensis*. De Oliveira et al. [216] reported that *T. harzianum* is recommended for the biological control of the root-lesion nematode *Pratylenchus brachyurus*, which is an important nematode in sugarcane, sunflower, potato, millet, cotton, corn, and soybean, while Wu et al. [217] found that octahydronaphthalene derivatives from an endophytic fungus *Trichoderma* sp. can be used for managing *Botrytis cinerea*. Tamandegani et al. [218] also found that *T. asperellum* (Iran 3062C), which was isolated from potato fields in Hamedan, Iran, showed a strong ability to induce systemic resistance against cucumber mosaic virus (CMV) that involved the jasmonic acid (JA)/ethylene (ET)/salicylic acid (SA) signaling pathways. Biocontrol mechanisms applied by the *Trichoderma* genus against fungal pathogens include the use of plant-resistance elicitors, the stimulation of antioxidative enzymes, and phytoalexin production by the plants via *Trichoderma* and its metabolites, as well as mechanisms like mycoparasitism, utilization of the activity of proteases, glucanases, and chitinases, competition for space and nutrients, and the production of antifungal and antibiotic components such as polyketides, peptaibols, anthraquinones, pyrones, and terpenoids.

*T. virens* 6PS-2 was effective in controlling apple replant disease and in improving the growth and fruit quality of apple [219], while *T. virens* could improve defense pathways [220] and enhance plant growth promotion in Arabidopsis when applied as an important biocontrol agent [220]. *T. harzianum* could increase the tolerance of avocado seedlings to *P. cinnamomic* [221], and it can be used as biological control agent to control post-harvest pathogens such as *Diaporthe* sp. and *Phomopsis perseae* in avocado plants [222]. *T. harzianum* also can significantly control chickpea *Fusarium* wilt [223,224]. It is also effective in the control of root-knot nematode disease in cowpea [225]. Gupta et al. [226] reported that *T. harzianum* can induce a higher root canopy and a better root biomass and final yield in finger millet. Its application was also effective against dry root rot in mung bean [227,228]. Pandey et al. [229] found that *T. harzianum* could enhance the activity of antioxidative enzymes and decrease lipid peroxidation during drought stress in rice plants. The application of an organic additive with *T. harzianum* could reduce the levels of *Sclerotium rolfsii*, which is the main pathogen for potato [230]. Limdolthamand et al. [231] could decrease the infection of northern corn leaf blight in sweet corn and improve the growth of sweet corn plants. *T. asperellum* could improve the resistance of plants against Colletotrichum graminicola by increasing lignification in plants and by enhancing the activity of antioxidative enzymes [232], and it was effective in controlling *Fusarium* wilt in stevia plants [233]. Changes in the yield and yield components of various horticultural and agricultural plants following the application of different species of *Trichoderma* spp. are presented in Table 3.

## 6. Conclusions

Endophytic fungi are very important in agricultural production because of their synergistic association with plants and their strong ability to stimulate plant growth because of their synthesis of phytohormones in response to biotic and abiotic stressors; these make them unique options as biofertilizers and biostimulants as well as as biocontrol factors against different types of diseases. Different species of *Penicillium* can significantly improve the fresh and dry weight of shoots, as well as stimulate the chlorophyll content. They can also improve the photosynthesis rate and the P and N content. *P. janthinellum* can be used to control phytophthora root rot in azalea, while *P. citrinum* YW322 can be applied to control ginseng root rot caused by *Fusarium*, and *P. citrinum* BTF08 can be applied against the pathogenic *F. oxysporum* s. sp. *cubense* race 4 in banana. *P. chrysogenum* can be used to control fungal diseases in cotton, and it is also appropriate for controlling root-knot nematodes in cucumber. *P. citrinum* can enhance the yield and chemical metabolites in choy sum, while *P. chrysogenum* strain 34-P can increase the fresh and dry biomass of maize, and *P. chrysogenum* (PenC-JSB9) is suitable for increasing the root and shoot length of pearl millet. Species of *Trichoderma* spp. are known as growth enhancers, stimulators of resistance in plants, biopesticides, and biofertilizers. *T. harzianum* is effective against dry root rot in mung bean, and *T. asperelloides* PSU-P1 can enhance the defense response against stem blight diseases in muskmelon. *T. viride* Tv-1511 can boost the concentrations of menthone, menthol, and pulegone in peppermint plants, while *T. asperellum* IIPRTH-31 and *T. afroharzianum* IIPRTH-33 can be used to control *Fusarium* wilt in pigeon pea plants. Among the different species, *T. viride*, *T. longibrachiatum*, *T. atroviride*, *T. hamatum*, and *T. koningii* have been reported to show nematicidal characteristics. *Trichoderma* spp. produces various secondary compounds with numerous beneficial impacts, such as xylanases, epipoly-thiodioxopiperazines, pyrones, peptaibols, volatile terpenes, nonvolatile terpenes, polyketides, siderophores, and cerato-plantanins. The most important impacts of *Trichoderma* inoculation are the destruction of pathogenic organisms and plant growth promotion. Their strains are considered to be among the most useful fungi in agriculture, horticulture, industrial enzyme production, and bioremediation. In conclusion, the application of *Penicillium* spp. and *Trichoderma* spp. is a promising method and an environmentally friendly practice for improving the growth and final yield of plants, and novel practices involving such biostimulants should be integrated in new farming systems. Areas that should be considered in future research include studying their mechanisms of action, assessing their long-term effects while considering sustainability and the effects of climate change, how to integrate both *Trichoderma* spp. and *Penicillium* spp. into sustainable agriculture, and investigating the effects of their combined application.

## Figures and Tables

**Table 1:** The effects of different species of *Penicillium* spp. on the yield and yield components of plants.

Plant	Plant Family	*Penicillium* spp. Species	Key Point	References
Azalea	Ericaceae	*P. janthinellum*	It can be used as a biological control agent of phytophthora root rot in azalea.	[97]
Banana	Musaceae	*P. citrinum* BTF08	It has great potential against the pathogenic *F. oxysporum* f. sp. *cubense* race 4 (FocR4)	[107]
Choy sum (*Brassica rapa* var. *parachinensis*)	Brassicaceae	*P. citrinum*	It can significantly increase yield and chemical metabolites.	[98]
Cotton (*Gossypium hirsutum* L.)	Malvaceae	Dry mycelium of *P. chrysogenum*	It is effective in controlling fungal diseases. It can be used against soil-borne pathogens that may penetrate the cotton root. It is important against wilt diseases.	[99]
		Dry mycelium of *P. chrysogenum*	It is effective in controlling *Verticillium dahliae* Kleb (*Vd*) and *F. oxysporum* f. sp *vasinfectum* (*Fov*) as well as the accumulation of pathogenesis-related protein transcripts.	[108]
			It is important in increasing resistance against *Verticillium dahliae* in cotton, which is the main cause of wilt in cotton.	[109]
Cucumber (*Cucumis sativus* L.)	Cucurbitaceae	*P. chrysogenum*	It can enhance the germination rate, germination index, and seed germination as well as increase the potential biocontrol against the root-knot nematode (*Meloidogyne incognita*) in cucumber.	[88]
Ginseng (*Panax ginseng* L.)	Araliaceae	*P. citrinum* YW322	It has special potency in controlling ginseng root rot disease caused by *F. oxysporum*.	[110]
Jujube (*Ziziphus jujuba* L.)	Rhamnaceae	*P. chrysogenum*	The endophyte *P. chrysogenum* has shown plant-growth-promoting activity and flowering-promoting activity.	[111]
Lettuce (*Lactuca sativa* L.)	Asteraceae	*P. pinophilum*	Inoculation with it can increase plant tolerance to *Rhizophagus intraradices* and *Penicillium pinophilum*.	[112]
Maize (*Zea mays* L.)	Poaceae	*P. chrysogenum* strain 34-P	Inoculation with it can influence maize seedling growth with significantly higher fresh biomass, dry biomass, and total chlorophyll.	[102]
Melon (*Cucumis melo* L.)	Cucurbitaceae	The extract of dead *P. chrysogenum*	It can enhance resistance against *Fusarium* wilt in melon.	[113]
Pearl millet (*Pennisetum glaucum* L.)	Poaceae	*P. chrysogenum* (PenC-JSB9)	It can positively promote growth, increase seed germination and shoot and root length, and induce resistance to downy mildew disease caused by *Sclerospora graminicola*.	[103]
Pineapple (*Ananas comosus* (L.) Merr.)	Bromeliaceae	*Penicillium* sp.	It can control pineapple internal browning. It can change the endophyte fungi community structure and abundance during storage. Exogenous inoculation may control internal browning by increasing the plant’s antioxidative capacity.	[114]
Pomegranate (*Punica granatum* L.)	Punicaceae	*P. pinophilum* (NFCCI2498)	Its inoculation into soil was found to promote nutrient uptake and induce a higher photosynthetic rate and leaf area index.	[115]
Rice (*Oryza sativa* L.)	Poaceae	*P. citreonigrum*	It can improve the yield and reduce the risk of harmful toxins.	[116]
Tobacco (*Nicotiana glutinosa* L.) (*Nicotiana benthamiana*) (*Nicotiana glutinosa*)	Solanaceae	Dry mycelium of *P. chrysogenum*	The crude peptides derived from it can be used to protect *Nicotiana glutinosa* against tobacco mosaic virus (TMV) by accelerating a TMV-N gene-triggered hypersensitive response (HR) rather than by priming callose deposition.	[117,118]
		*P. chrysogenum*	It can be used to prevent TMV spread in *N. benthamiana* plants.	[119]
		Dry mycelium of *P. chrysogenum*	It can activate defense responses in tobacco BY-2 cell suspensions, including the expression of defense responses genes and the accumulation of secondary metabolites against TMV.	[120]
		*P. chrysogenum*	It can protect flue-cured tobacco against brown spot and wildfire disease.	[121]
		Dry mycelium of *P. chrysogenum*	The abscisic acid biosynthetic pathway and β-1,3-glucanase, a callose-degrading enzyme, have significant roles in enhancing the defense response against TMV.	[122]
		Dry mycelium of *P. chrysogenum*	A polypeptide and a polysaccharide extract of it can induce the production of hydrogen peroxide and nitric oxide and improve the resistance of Peruvian tobacco (*Nicotiana glutinosa*) to TMV.	[123]
Tomato (*Solanum lycopersicum* L.)	Solanaceae	Dry mycelium of *P. chrysogenum*	It can promote resistance in plants against the root-knot nematode *Meloidogyne javanica*.	[104]
		*P. janthinellum* LK5	It can modulate stress responses through a reduction in the level of jasmonic acid and the synthesis of ABA. It can increase the resistance of plants to salinity stress by activating defensive mechanisms and the production of gibberellins in the hosts.	[105]
		*P. janthinellum* LK5 (PjLK5)	Its application could enhance metal phytoextraction while promoting crop physiological homeostasis.	[106]
Wheat (*Triticum aestivum* L.)	Poaceae	*P. chrysogenum* #5TAKL-3a	It is an appropriate substitute that can be used to increase the adaptability of plants under drought stress. It can also increase the resistance of plants to drought stress by increasing the production of ammonia, gibberellic acid, and IAA.	[124]

**Table 2:** Occurrence of *Trichoderma* spp. in different environments.

Environment	*Trichoderma* spp. Species	References
Desert soil	*T. afroharzianum*, *T. atrobrunneum*, *T. longibrachiatum*	[175]
Forest soil	*T. afroharzianum*, *T. asperellum*, *T. atroviride*, *T. citrinoviride*, *T. fertile*, *T. harzianum*, *T. longibrachiatum*, *T. oblongisporum*, *T. pararogersonii*, *T. paraviridescens*, *T. pleurotum*, *T. piluliferum*, *T. polysporum*, *T. rossicum*, *T. saturnisporum*, *T. viridescens*	[176,177]
Soil from garlic crops	*T. asperelloides*, *T. azevedoi*, *T. hamatum*, *T. koningiopsis*, *T. longibrachiatum*, *T. peberdy*	[178]
Soil from maize crops	*T. asperellum*, *T. brevicompactum*, *T. fertile*, *T. hamatum*, *T. harzianum*, *T. koningiopsis*, *T. longibrachiatum*, *T. pleuroticola*, *T. virens*	[179]
Soil from onion crops	*T. afroharzianum*, *T. asperelloides*, *T. asperellum*, *T. azevedoi*, *T. erinaceum*, *T. lentiforme*, *T. longibrachiatum*, *T. peberdyi*	[178]
Soil from rice crops	*T. asperellum*, *T. atroviride*, *T. brevicompactum*, *T. capillare*, *T. erinaceum*, *T. fertile*, *T. hamatum*, *T. harzianum*, *T. koningiopsis*, *T. longibrachiatum*, *T. polysporum*, *T. saturnisporum*, *T. spirale*, *T. velutinum*, *T. virens*	[179]
Soil from wheat crops	*T. asperellum*, *T. atroviride*, *T. brevicompactum*, *T. erinaceum*, *T. hamatum*, *T. harzianum*, *T. koningiopsis*, *T. virens*	[179]
Soil from plant roots	*T. afroharzianum*, *T. asperelloides*, *T. asperellum*, *T. guizhouense*, *T. harzianum*, *T. reesei*, *T. strigosum*, *T. virens*	[180]
Soil from tree bark	*T. atroviride*, *T. erinaceum*, *T. harzianum*, *T. hebeiensis*, *T. longibrachiatum*, *T. parareesei*, *T. reesei*	[181]
From decaying wood	*T. atroviride*, *T. citrinoviride*, *T. cremeum*, *T. gamsii*, *T. harzianum*, *T. koningii*, *T. koningiopsis*, *T. longibrachiatum*, *T. longipile*, *T. paraviridescens*, *T. trixiae*, *T. viride*, *T. viridescens*	[182]
From lignocellulosic compost	*T. asperellum*, *T. citrinoviride*, *T. harzianum*, and *T. lixii*	[183,184]

**Table 3:** The effects of different species of *Trichoderma* spp. on the yield and yield components of some agricultural and horticultural plants.

Plant	Plant Family	*Trichoderma* Species	Key Points	References
Apple (*Malus robusta*)	Rosaceae	*T. virens* 6PS-2	It was effective in controlling apple replant disease (ARD) and improved the growth and final quality of fruits.	[234]
		*T. asperellum*	Significant reduction in the negative impacts caused in the host by *F. proliferatum* was reported.	[234]
Arabidopsis (*Arabidopsis thaliana*)	Brassicaceae	*T. virens*	It could secrete 1-octen-3-ol, which enhanced plant resistance against pathogens by activating JA/ET-dependent defense pathways.	[235,236]
		*T. virens*	It could be used for biocontrol goals, balancing between herbicidal activity and plant-growth promotion.	[220]
Avocado (*Persea americana* Mill)	Lauraceae	*T. harzianum*; *T. hamatum*	They could increase the tolerance of plants to *P. cinnamomi*.	[221]
		*T. harzianum*	It could be used as a biological control agent to suppress post-harvest pathogens like *Phomopsis perseae*, *Diaporthe* sp., *Colletotrichum gloeosporioides*, and *Neofusicoccum parvum*.	[237]
		*T. atroviride*; *T. virens*	They could promote the control of avocado white root rot.	[238]
Banana (*Musa* sp. L.)	Musaceae	*T. harzianum* ALL42 and IBLF006; *T. asperellum* T00	They could promote plant growth and reduce the negative impacts of *Meloidogyne javanica*.	[239,240]
		*T. longibrachiatum*	It could promote the performance of banana seedlings.	[241]
		*T. asperellum*	Inoculation with it could increase the root potassium and phosphorus content.	[242]
		*T. piluliferum*	It is an important biocontrol agent for anthracnose caused by *Colletotrichum musae*.	[243]
Bean (*Phaseolus vulgaris* L.)	Fabaceae	*T. harzianum*	It can induce plant resistance to *R. solani* by increasing the synthesis of squalene and ergosterol and by promoting the stability and integrity of plant cell membranes.	[244,245,246]
		*T. viride*	It can enhance the levels of total phenols, polyphenol, peroxidase, phenylalanine ammonia-lyase, and photosynthetic pigments and protect plants against fungal infections.	[247]
		*T. longibrachiatum* AD-1	It can be used to control root rot diseases caused by *Fusarium* spp. RT-qPCR revealed that applying a cell-free culture filtrate of *Trichoderma* in common beans resulted in significant up-regulation of the defense-related genes PR1, PR2, PR3, and PR4.	[248]
		*T. gamsii* IT-62	It could lead to increased soil fertility and significantly improve the root and shoot weight and levels of total protein, and photosynthetic pigments.	[249]
		*T. gamsii* IT-62	It can be used to control common bean damping-off caused by pathogens.	[250]
		*T. asperellum* BRM-29104	It can be used against white mold caused by *Sclerotinia sclerotiorum*.	[251]
Black pepper (*Piper nigrum* L.)	Piperaceae	T. *viride*; *T. harzianum*	They can significantly protect black pepper plants from anthracnose by increasing the chlorophyll content, increasing the overall plant health, suppressing the disease incidence and improving the innate defense system and plant health.	[252,253,254,255]
Cacao (*Theobroma cacao* L.)	Malvaceae	*T. asperellum* PR11	It could be used against cacao black pod rot (BPR) caused by *Phytophthora megakarya*.	[256,257,258]
Canola (*Brassica napus* L.)	Brassicaceae	*T. harzianum* (ARCTr281, ARCTr272, and ARCTr418)	They were effective antagonists in controlling canola rot disease and increasing plant traits.	[259]
Cassava (*Manihot esculenta* Crantz)	Euphorbiaceae	*T. aureoviride* URM5158	It could manage cassava root rot.	[260]
Castor (*Ricinus communis* L.)	Euphorbiaceae	*T. asperellum* 7316, *T. asperellum* N13	They showed special antagonistic ability against the fungal pathogen (*Alternaria ricini*).	[261,262]
Chickpea (*Cicer arietinum* L.)	Fabaceae	*T. harzianum*	It was effective in the control of chickpea *Fusarium* wilt.	[223]
		*T. harzianum*	It could reduce the adverse impacts of salt stress and fungal diseases.	[224]
		*T. harzianum*; *T virens*	They could increase root and shoot lengths and grain yield as well as reduce the dry root rot incidence.	[263]
Chili (*Capsicum frutescens* L.)	Solanaceae	*T. asperellum*; *T. harzianum*; *T virens*; *T. pseudokoningii*	They could increase the resistance of plants to *Colletotrichum truncatum* by enhancing the activity of defensive and antioxidative enzymes in plants, decreasing the accumulation of reactive oxygen in plants.	[264]
Chinese cabbage (*Brassica rapa* L.)	Brassicaceae	*Trichoderma* spp.	They could increase nutrient uptake and increase tolerance to environmental stresses as well as improve the quality of products.	[265]
Citrus (*Citrus* L.)	Rutaceae	*T. harzianum*	It can affect *Guignardia citricarpa* and deactivate the hydrolytic enzymes of pathogens.	[266]
Cacao tree (*Theobroma cacao* L.)	Malvaceae	*T. hamatum*	It could increase stomatal conductance, green fluorescence emission, net photosynthesis, and improve drought tolerance.	[267]
Coffee (*Coffea* L.)	Rubiaceae	*T. asperellum* GD040	It is suggested for managing coffee anthracnose caused by *Colletotrichum cairnsense*.	[268]
Cotton (*Gossypium hirsutum* L.)	Malvaceae	*T. virens*	It can increase terpenoid synthesis and is toxic to *R. solani.*	[269]
		*T. virens* and *T. longibrachiatum*	They could metabolize pathogen propagule germination against *R. oryzae*.	[270,271]
		*T. hamatum*	It has a great capacity to control leaf worm (*Spodoptera littoralis*).	[272]
		*T. viride*	It decreased the root rot incidence and improved yield and dry matter production.	[273]
		*T. viride* NBAITv23; T. *harzianum* NBAIITh1	Because of their antifungal activities, they can be used against *R. solani*, which is the cotton seed rotting pathogen.	[274]
Cowpea (*Vigna unguiculata* L.)	Fabaceae	*T. harzianum*	It was effective in the control of root-knot nematode disease.	[275]
Cucumber (*Cucumis sativus* L.)	Cucurbitaceae	*T. atroviride*	It can lead to resistance to *R. solani* by increasing the accumulation of volatile organic compounds, increasing the activity of antioxidative enzymes in the plant, and reducing lipid peroxidation.	[276,277]
		*T. longibrachiatum*	It can increase plant resistance against *Botrytis cinerea* by increasing the contents of jasmonic acid, salicylic acid, and ethylene.	[278]
		*T. asperellum*	In high-salinity conditions, it could increase the level of abscisic acid, auxins, and gibberellin.	[279]
		*T. asperellum*	It could enhance local defense responses and plant root colonization with the secretion of swollenin.	[280]
		*T. asperellum* FJ035	It was effective against cucumber *Fusarium* wilt and improved the plant height, root length, fresh weight, stem thickness, and quality.	[281]
		*T. asperellum*; *T. pseudokoningii*	They have shown high potential as biocontrol fungi against cucumber *Fusarium* wilt.	[282]
		*T. koningiopsis*	An alternative bioherbicide against different fungi of the cucumber plants due to its ability to produce enzymes that increase its phytotoxic effects.	[283]
Dragon fruit (*Hylocereus costaricensis*)	Cactaceae	*T. asperellum* K1-02	It has shown antifungal activity against stem canker caused by the fungus *Neoscytalidium dimidiatum*.	[284]
*Eucalyptus* trees (*Eucalyptus globulus*)	Myrtaceae	*T. asperellum*; *T. longibrachiatum*	They could effectively suppress the growth of *Neofusicoccum parvum* and *Lasiodiplodia theobromae* fungal species.	[285]
Faba bean (*Vicia fabae* L.)	Fabaceae	*T. viride*, *T. harzianum*, *T. virens*, *T. atroviride*, *T. longibrachiatum*	They can be used to reduce the growth of *Alternaria* spp., *R. solani*, *Botrytis* spp., *Penicillium* spp., *Aspergillus* spp., *F. solani*, and *F. oxysporum*.	[286,287]
Finger millet (*Eleusine coracana* L.)	Poaceae	*T. harzianum*	Its application induced a higher root canopy and a better root biomass and final yield.	[288]
Forage cactus (*Nopalea cochenillifera* L.)	Cactaceae	*Trichoderma* spp.; *T. aureoviride* URM 6668	They have shown high efficiency against the soil fungi *F. solani* and *Lasiodiplodia theobromae*.	[289]
Forskolin (*Coleus forskohlii* (Willd.) Briq.)	Lamiaceae	*T. viride*	Its inoculation could enhance the root biomass and forskolin content and reduce nematode infections.	[290]
Grapevine (*Vitis vinifera* L.)	Vitaceae	*T. koningiopsis*	It could be used to reduce copper levels and fight against *Plasmopara viticola*, the causal agent of downy mildew.	[291]
		*T. harzianum*	It has positive impacts in controlling soil-borne plant pathogens like *F. solani*, *F. oxysporum*, and *Macrophomina phaseolina*.	[292]
		*T. simmonsii*; *T. afroharzianum*; *T. gamsii*; *T. orientale*	They could significantly improve D-glucose and the D-fructose concentrations.	[293]
		*T. harzianum* T118	Its application induced higher total antioxidative activity and levels of ascorbate peroxidase and flavonoids.	[294]
Groundnut (*Arachis hypogaea* L.)	Fabaceae	*T. viride JAU60*	It can be used to combat the oxidative burst produced by invading pathogens, especially against *Aspergillus niger* Van Tieghem, which causes collar rot.	[295,296]
		*T. longibrachiatum*; *T. asperellum*	They showed an important role in the resistance to *Sclerotium rolfsii*.	[297]
		*T. asperellum* SH16	It was important in controlling diseases such as root rot, stem rot, and pod rot.	[298]
		*T. asperelloides* SKRU-01	It can be used for mitigating the negative impacts of *Aspergillus* species infestation and for increasing aflatoxin B_1_ degradation.	[299]
		*T. harzianum* ITEM 3636	It could be used against *F. solani* and reduced the severity of brown root rot.	[300,301]
		*T. viride* F7	It could enhance peanut performance and decrease peanut Cd concentrations.	[302]
Jerusalem artichoke (*Helianthus tuberosus* L.)	Asteraceae	*T. harzianum*	It is suggested for the biological control of southern stem rot caused by *Sclerotium rolfsii*.	[303]
Lettuce (*Lactuca sativa* L.)	Asteraceae	*T. azevedoi* CEN1241	It could increase the levels of volatile organic compounds and decreased the negative impacts of white mold caused by the fungus *Sclerotinia sclerotiorum*.	[304]
		*T. asperellum* T76-1	It could inhibit fungal growth of two leaf-spot fungal pathogens, namely *Curvularia aeria* and *Corynespora cassiicola*.	[305]
		*T. spirale* T76-1	It could decrease the native effects of leaf spot caused by *Curvularia aeria* and *Corynespora cassiicola*.	[306]
		*T. harzianum* T22; *T. atroviride* P1	They could alleviate abiotic stresses and improved plant growth.	[307]
Mango (*Mangifera indica* L.)	Anacardiaceae	*T. asperellum* T8a	It can enhance the control of anthracnose via cellulase activity as well as mango production.	[308]
		*T. pinnatum* LS029-3	It could improve the activity of mango defense enzymes such as total phenol, peroxidase, and polyphenol oxidase and reduced the content of ascorbic acid content and glutathione enzyme, and thus be useful against mango stem-end rot disease.	[309]
Maize (*Zea mays* L.)	Poaceae	*T. viride*; *T. harzianum*	They are effective against *F. verticillioides* and *F. proliferatum* strains.	[310,311]
		*T. asperelloides*; *T. longibrachiatum*	These two species increase seedlings’^,^ wet biomass and increase the resistance of plants against late wilt.	[312]
		*T. harzianum*	It elicited plant defense responses through the secretion of Hyd1 hydrophobin.	[313]
		*T. harzianum*	It induced ISR in plants via ET or JA pathways via the secretion of cellulases.	[314]
		*T. asperellum* GDFS1009	It could significantly reduce maize stalk rot caused by *F. graminearum*.	[315]
		*T. gamsii*	It could improve plant growth and increase the resistance of plants to *F. verticillioides*.	[316]
Milk thistle (*Silybum marianum* (L.) Gaertn.)	Asteraceae	*Trichoderma* strain M7, KHB, G124-1, G46-3, and G46-7	They could promote growth and increased the production of silymarin, isosilybin, and silychristin.	[317]
Monterey pine (*Pinus radiata*)	Pinaceae	*T. hamatum*	Its application in seedlings and roots could increase the dry root weight, shoot growth promotion, and root penetration.	[318,319]
Mulberry (*Morus* spp.)	Moraceae	*T. pseudokoningii*	It could decrease the negative impacts of soil-borne diseases and improved plant growth.	[266]
Mung bean (*Vigna radiata* L.)	Fabaceae	*T. harzianum*	It was effective against dry root rot.	[228]
Muskmelon (*Cucumis melo* L.)	Cucurbitaceae	*T. asperelloides PSU*-P1	It revealed antifungal characteristics and increased the defense response against gummy stem blight through cell-wall-degrading enzymes and defense-related enzymes.	[320]
Mustard (*Brassica juncea* L.)	Brassicaceae	*T. viride* 1433 mutant strains	They could improve the tolerance of plants to *Pythium aphanidermatum* in both field and lab experiments.	[321]
Oil palm (*Elaeis guineensis*)	Palmaceae	*T. asperellum* T76-14	It is an important choice to improve plants against *Ganoderma boninense* infection.	[322]
Olive (*Olea europaea* L.)	Oleaceae	*T. harzianum strain Ths97*	It could increase plant tolerance to *F. solani*.	[323]
Onion (*Allium cepa* L.)	Amaryllidaceae	*T. viride*	The combined application of *T. viride* and AMF altered amino acid concentrations.	[324]
		*T. viride*	The combined application of *T. viride* and AMF significantly improved the levels of total free amino acids, the soluble protein content, and onion biomass.	[324]
		*T. asperellum*	In the presence of heavy metals, it could decrease lipid peroxidation and regulate the level of proline in the plants.	[325]
		*T. longibrachiatum*	It could decrease electrolyte levels elevated by infection and salinity and decrease the damage caused by infection with *Sclerotium cepivorum*.	[326]
		*T. azevedoi* CEN1241; *T. koningiopsis* CEN1513; *T. asperelloides* CEN1559	They could increase the weight of onion bulbs promote seedling growth, and be useful against soil fungi, especially *Sclerotium rolfsii*.	[327]
		*T. harzianum*; *T. atroviride*	They could mitigate the adverse effects of *Fusarium* basal rot caused by *F. oxysporum* f. sp. cepae.	[328]
Pak Choi (*Brassica campestris* spp. *Chinensis*)	Brassicaceae	*T. viride*	Its inoculation can increase plant growth, with significant impacts on suppressing clubroot disease (*Plasmodiophora brassicae*).	[329]
		*T. harzianum*	It could boost plant growth and the activity of antioxidative enzymes such as peroxidase, glutathione reductase, catalase, superoxide dismutase, and ascorbic acid peroxide.	[330]
Pearl millet (*Pennisetum glaucum* (L.) R.Br.)	Poaceae	*T. virens*	Its oligosaccharides can increase resistance against downy mildew disease.	[331]
		*T. asperellum* DL-81	It is suggested as an important biological control agent of downy mildew disease.	[332]
Pepper (*Capsicum annuum* L.)	Solanaceae	*T. polysporum T1*	It was effective in the control of leaf curl virus (*PeLCV*) by releasing salicylic acid.	[333]
		*T. atroviride* T32l; *Trichoderma* sp. N97	Sustainable candidates for the biological control of root rot pathogen and pepper wilt.	[334,335]
		*T. asperellum*	It has a positive effect in controlling root-knot nematodes arising from *Meloidogyne incognita.*	[336]
		*T. virens* HZA14	It could reduce the disease incidence and delayed the occurrence of chili pepper blight caused by *Phytophthora capsici*.	[337]
		*T. simmonsii*	It could increase crop nutrition and stimulated the plant tolerance to *P. capsici*.	[338]
Peppermint (*Mentha* × *piperita* L.)	Lamiaceae	*T. viride* Tv-1511	Its inoculation can enhance concentrations of pulegone, menthol, and menthone.	[339]
Pigeon pea (*Cajanus cajan* L.)	Fabaceae	*T. asperellum* IIPRTH-31; *T. afroharzianum* IIPRTh-33	They could be effective in the biocontrol of *Fusarium* wilt through defense-related enzymes.	[340]
		*T. harzianum*; *T. viride*	They could increase growth and resistance against *Fusarium* sp.	[341]
Pine (*Pinus sylvestris*)	Pinaceae	*T. harzianum*	It could increase plant tolerance to *Phytophthora cinnamon* under greenhouse conditions.	[342,343]
Pomegranate (*Punica granatum* L.)	Punicaceae	*Trichoderma* spp. (strains ABSA18, TSA17, and ABSA16)	They were effective against pathogenic fungi of pomegranate such *F. chlamydosporum*, *F. oxysporun*, *A. niger*, and *A. alternata*.	[344]
Potato (*Solanum tuberosum* L.)	Solanaceae	*Trichoderma* spp.	It could be used as an important alternative against *Alternaria* solani, which is the main pathogen causing early blight in potato.	[345]
		*T. brevicompactum*	It was effective in the biological control of potato wilt disease (*F. solani*).	[346]
Rambutan (*Nephelium lappaceum* L.)	Sapindaceae	*T. harzianum*	It could reduce the occurrence of post-harvest diseases caused by pathogens such as *Botryodiplodia theobromae*, *Colletotrichum gloeosporioides*, *Gliocephalotrichum microchlamydosporum* and improve the color and quality of the fruits.	[347]
Rice (*Oryza sativa* L.)	Poaceae	*T. harzianum*	It could increase the activity of antioxidative enzymes and reduce lipid peroxidation during drought stress.	[348]
		*T. hamatum* KUFA 0042	It showed high biocontrol activity against *R. solani* and *Bipolaris oryzae*.	[349]
		*T. asperellum* T12	It was effective in the biocontrol of sheath blight.	[350]
Roselle (*Hibiscus sabdariffa* L.)	Malvaceae	*T. viride*	It could reduce the mycelial growth of different pathogens such as *R. solani*, *F. nygamai*, and *Phoma exigua*.	[351]
Rye (*Secale cereale* L.)	Poaceae	*T. harzianum*	It could increase the plant tolerance to *R. solani* and *Sclerotium rolfsii* under both field and greenhouse conditions.	[352]
		*Trichoderma* spp.	They have a positive influence on the growth and final yield of plants.	[353]
Ryegrass (*Lolium multiflorum* L.)	Poaceae	*T. atroviride*	It could promote resistance against *Pyricularia oryzae* in ryegrass through physical and biochemical defenses.	[354]
Soybean (*Glycine max* L.)	Fabaceae	*T. viride*	It can be used as a biocontrol agent against two fungal pathogens, namely *Pythium arrhenomanes* and *F. oxysporun*. It could promote the root system and enhance the root and shoot systems.	[355]
		*Trichoderma* strains	They have shown positive effects against charcoal rot caused by *Macrophomina phaseolina* (Tassi) Goid.	[272]
		*Trichoderma* spp.	They could improve the absorption of Mn and K and reduce the severity and incidence of diseases.	[356]
		*T. koningiopsis*	It is an important alternative for weed control.	[357]
		*Trichoderma* spp.	Trichoderma-based products could control the spiral nematode (*Helicotylenchus dihystera*).	[358]
		*T. harzianum*; *T. viride*; *T. koningii*	They could significantly inhibit the mycelial growth of *F. oxysporum*, *F. solani*, and *R. solani*.	[359]
		*T. harzianum* ALL 42	It has shown special potential in the biological control of *Pratylenchus branchyrus*, an important nematode.	[275]
Sorghum (*Sorghum bicolour* L.)	Poaceae	*T. asperellum*	It can improve the resistance of plants to *Colletotrichum graminicola* by increasing lignification in plants and improving the activity of antioxidative enzymes.	[232]
Spinach (*Spinacia oleracea* L.)	Amaranthaceae	*T. harzianum*	It could reduce the negative impacts of salt stress and improve the fresh and dry weight, root length, chlorophyll content, and mineral contents.	[360]
Stevia (*Stevia rebaudiana*)	Asteraceae	*T. asperellum*	It was effective in fighting against *Fusarium* wilt.	[233]
Strawberry (*Fragaria* × *ananassa* Duch.)	Rosaceae	*T. atrobrunneum*	It could increase plant tolerance to *Armillaria mellea*.	[361]
		*T. harzianum*	It was appropriate for preventing and controlling gray mold in plants.	[362]
Sugar beet (*Beta vulgaris* spp. *vulgaris*)	Amaranthaceae	*T. atroviride*	It can induce stress-related defense genes, such as the expression of a pathogenesis-related gene (PR-3), in plants.	[363]
		*T. viride* TVB1	It could decrease root rot disease and improve root yield.	[364]
		*T. harzianum*	It could reduce the incidence of *Sclerotium* root rot and enhanced the green foliage, sucrose yield per ha, and the root yield.	[365]
		*T. harzianum*; *T. asperellum*	They can be considered potential biocontrol agents for controlling *R. solani*-induced sugar beet damping-off disease.	[366]
Sugarcane (*Saccharum officinarum* L.)	Poaceae	*T. harzianum*	It could increase soil carbon sequestration, photosynthesis, yield, and growth in sugarcane ratoon.	[367]
Sunflower (*Helianthus annuus* L.)	Asteraceae	*T. longibrachiatum*	It could increase the activity of antioxidative enzymes in plants in the presence of heavy metals.	[368,369]
		*T. harzianum* T22	It could increase the tolerance of plants to *Alternaria alternata* in lab experiments.	[370]
		*T. viride*	Its application together with *P. fluorescens* could increase the oil content and growth of plants.	[371]
		*T. harzianum* T22	It could decrease sclerotia formation caused by *Sclerotinia sclerotiorum* in sunflower.	[372]
		*T. harzianum* TRIC8	It was effective in improving the resistance of plants to downy mildew disease caused by *Plasmopara halstedii*.	[373]
Sweet sorghum (*Sorghum bicolor* (L.) Moench)	Poaceae	*T. viride*	It had an important role in reducing NH_3_ volatilization and simultaneously improved the effectiveness of nitrogen fertilizers.	[374]
Sweet corn (*Zea mays* convar. saccharata var. rugosa)	Poaceae	*T. harzianum*	It reduced the infection of northern corn leaf blight in sweet corn as well as improved plant growth.	[375]
Sword lily (*Gladiolus hybridus*)	Iridaceae	*T. hamatum*	It could increase chlorophyll a and b levels and the uptake of both macro and micronutrients and enhanced inflorescence elongation.	[376]
Tea (*Camellia sinensis* L.)	Theaceae	*T. reesei* TRPATH01	It could increase the shoot height, stem diameter, and fresh weight and improved the resistance of plants to *Fusarium* dieback caused by *F. solani*.	[377]
		*T. reesei*	It can be considered for the biological control of gray blight.	[348]
		*T. asperellum* TC01	It could be applied to fight against *Colletotrichum gloeosporioides* C62. It could increase the root dry weight, shoot dry weight, stem diameter, and shoot height.	[348]
Tobacco (*Nicotiana tabacum* L.)	Solanaceae	*T. nigricans* T32781	It could result in lower Cd uptake and contamination in plants and improved tobacco growth.	[378]
Tomato (*Solanum lycopersicum* L.)	Solanaceae	*T. viride*	Its inoculation could significantly increase the root fresh weight and shoot fresh weight. Its application was more effectual in controlling early blight disease caused by *Alternaria alternata*.	[379,380]
		*T. harzianum*	It could improve the final yield and inhibited the radial growth of *F. oxysporum* f. sp. *lycopersici*.	[381,382,383]
		*T. harzianum*; *T. viride*	Both of them could be used against root galling and the nematode reproduction of *Meloidogyne javanica*.	[384,385,386]
		*T. harzianum*; *T. viride*	They could promote growth and increase the yield of tomato plants.	[384]
		*T. harzianum* TR05; *T. viride* TR06; *T. asperellum* TR08	They have been suggested as biological control agents of collar rot.	[387]
		*T. hamatum*	Its application on seedlings could enhance lateral development.	[388]
		*T. simmonsii*; *T. atrobrunneum*	They are effective in suppressing disease development and colony growth of soil-borne pathogens.	[389,390]
		*T. koningii*	It could increase the activity of antioxidative enzyme in plants at high temperatures as well as regulate the level of starch, proline, phenols, and proteins.	[391]
		*T. harziaum*	At low temperatures, it could decrease lipid peroxidation, regulate the level of osmolites, and increase the water content of the leaves.	[391]
		*T. harziaum*	During drought stress, it could increase the level of abscisic acid, gibberellin, and auxins in the plant.	[392]
		*T. asperellum*	It is promising for the successful management of collar rot disease (*Agroathelia rolfsii*).	[393,394]
		*T. harziaum*; *T. atroviride*	They could secret 6PP, which can increase the leaf area and plant height, develop the root system, and enhance the lycopene content in fruits.	[395,396]
		*T. longibrachiatum*	It could influence genes involved in the mitigation of stress damage.	[397]
Tuberous begonias (*Begonia* × *tuberhybrida*)	Begoniaceae	*T. hamatum*	Its application to root tubers could increase the blooming size of the flowers and chlorophyll production and promote the uptake of boron, iron, and zinc.	[398]
Water hyssop (*Bacopa monnieri* L.)	Plantaginaceae	*T. harzianum*; *T. asperellum*	Their application was effective against *Alternaria alternata* causing leaf blight.	[399]
Wheat (*Triticum aestivum* L.)	Poaceae	*T. longibrachiatum*	It could increase the level of salicylic acid, reduce the level of hydrogen peroxide, and improve the activity of antioxidative enzymes in plants under salinity conditions.	[400]
		*T. harzianum sensu lato* TSM39	It is a potential biocontrol agent against *Bipolaris sorokiniana*, the causal agent of wheat spot blotch.	[400]
		*T. harzianum*; *T. pseudokoningii*	They have been introduced as having herbicidal potential in the control of *Rumex dentatus* L.	[401]
		*T. gamsii* MK361 138	It could reduce the severity of crown rot disease and improve growth parameters.	[402]
		*T. harzianum*; *T. viride*	They showed higher efficiency against *Alternaria alternata*, which can cause black point disease in wheat.	[403,404]
		*T. atroviride* Vel1	It is involved in mycoparasitism, sporulation, secondary metabolite production, and disease control and is especially appropriate for controlling the wheat root rot disease caused by *F. graminearum*.	[405]
Wild blueberries (*Vaccinium angustifolium*)	Ericaceae	*T. harzianum* T-22; *T. atroviride* IC-11	Its combination with calcium polysulphide could manage *botrytis* blossom blight in wild blueberry.	[406,407]

## Data Availability

Not applicable.

## References

[1] Shahrajabian M.H., Chaski C., Polyzos N., Tzortzakis N., Petropoulos S.A. (2021). Sustainable agriculture systems in vegetable production using chitin and chitosan as plant biostimulants. Biomolecules.

[2] Shahrajabian M.H., Chaski C., Polyzos N., Petropoulos S.A. (2021). Biostimulants application: A low input cropping management tool for sustainable farming of vegetables. Biomolecules.

[3] Shahrajabian M.H., Sun W., Cheng Q. (2022). Using bacteria and fungi as plant biostimulants for sustainable agricultural production systems. Recent Pat. Biotechnol..

[4] Sun W., Shahrajabian M.H. (2022). The effectiveness of rhizobium bacteria on soil fertility and sustainable crop production under cover and catch crops management and green manuring. Not. Bot. Horti Agrobot. Cluj Napoca.

[5] Chabbi N., Chafiki S., Telmoudi M., Labbassi S., Bouharroud R., Tahiri A., Mentag R., El-Amri M., Bendiab K., Hsissou D. (2024). Plant growth promoting rhizobacteria improve seeds germination and growth of *Argania spinosa*. Plants.

[6] Raish S.M., Sourani O.M., Abu-Elsaoud A.M. (2025). Plant growth-promoting microorganisms as biocontrol agents: Mechanisms, challenges, and future prospects. Appl. Microbiol..

[7] Embacher J., Seehauser M., Kappacher C., Stuppner S., Zeilinger S., Kirchmair M., Neuhauser S. (2023). *Serpula lacrymans* reacts with a general, unspecialized chemical response during interaction with mycoparasitic *Trichoderma* spp. and bacteria. Fungal Ecol..

[8] Shahrajabian M.H., Cheng Q., Sun W. (2022). The effects of amino acids, phenols and protein hydrolysates as biostimulants on sustainable crop production and alleviated stress. Recent Pat. Biotechnol..

[9] Shahrajabian M.H., Petropoulos S.A., Sun W. (2023). Survey of the influences of microbial biostimulants on horticultural crops: Case studies and successful paradigms. Horticulturae.

[10] Kim K., Lee Y., Ha A., Kim J.-I., Park A.R., Yu N.H., Son H., Choi G.J., Park H.W., Lee C.W. (2017). Chemosensitization of *Fusarium graminearum* to chemical fungicides using cyclic lipopeptides produced by *Bacillus amyloliquefaciens* strain JCK-12. Front. Plant Sci..

[11] De Souza R.R., Moraes M.P., Paraginski J.A., Moreira T.F., Bittencourt K.C., Toebe M. (2022). Effects of *Trichoderma asperellum* on germination indexes and seedling parameters of lettuce cultivars. Curr. Microbiol..

[12] Kumar K., Thakur P., Rathore U.S., Kumar S., Mishra R.K., Amaresan N., Pandey S., Mishra M. (2022). Plant beneficial effects of *Trichoderma* spp. suppressin *Fusarium* wilt and enhancing growth in tomato. Vegetos.

[13] Sun W., Shahrajabian M.H. (2023). The application of arbuscular mycorhhizal fungi as microbial biostimulant, sustainable approaches in modern agriculture. Plants.

[14] Sun W., Shahrajabian M.H., Petrpoulos S.A., Shahrajabian N. (2023). Developing sustainable agriculture systems in medicinal and aromatic plant production by using chitosan and chitin-based biostimulants. Plants.

[15] Ma H.-G., Liu Q., Zhu G.-L., Liu H.-S., Zhu W.-M. (2016). Marine natural products sourced from marine-derived *Penicillium fungi*. J. Asian Nat. Prod. Res..

[16] Nobre C., Nascimento A.K.C.D., Silva S.P., Coelho E., Coimbra M.A., Cavalcanti M.T.H., Teixeira J.A., Porto A.L.F. (2019). Process development for the production of prebiotic fructo-oligosaccarides by *Penicillium citreonigrum*. Bioresour. Technol..

[17] Huang J.-N., Zou Q., Chen J., Xu S.-H., Luo D., Zhang F.-G., Lu Y.-Y. (2018). Phenols and diketopiperazines isolated from Antarctic-derived fungi, *Penicillium citreonigrum* SP-6. Phytchem. Lett..

[18] Huber A., Galgoczy L., Varadi G., Holzknecht J., Kakar A., Malanovic N., Leber R., Kochf J., Kellerf M.A., Battag G. (2020). Two small, cysteine-rich and cationic antifungal proteins from *Penicillium chrysogenum*: A comparative study of PAF and PAFB. Biochim. Biophys. Acta-Biomembr..

[19] Zhao X., Liu X., Zhao H., Ni Y., Lian Q., Qian H., He B., Liu H., Ma Q. (2021). Biological control of *Fusarium* wilt of sesame by *Penicillium bilaiae* 47M-1. Biol. Control.

[20] De Cal A., Sztejnberg A., Sabuquillo P., Melgarejo P. (2009). Management *Fusarium* wilt on melon and watermelon by *Penicillium oxalicum*. Biol. Control.

[21] Waqas M., Khan A.L., Kamran M., Hamayun M., Kang S.M., Kim Y.H., Lee I.J. (2012). Endophytic fungi produce gibberellins and indoleacetic acid and promotes host-plant growth during stress. Molecules.

[22] Fan Y., Luan Y., An L., Yu K. (2008). Arbuscular mycorrhizae formed by *Penicillium pinophilum* improve the growth nutrient uptake, and photosynthesis of strawberry with two inoculum-types. Biotechnol. Lett..

[23] Maccari G., Deodato D., Fiorucci D., Orofino F., Truglio G.I., Pasero C., Martini R., Luca F.D., Docquier J.-D., Botta M. (2017). Design and synthesis of a novel inhibitor of *T. viride* chitinase through an *in silico* target fishing protocol. Bioorg. Med. Chem. Lett..

[24] Jia M., Chen J., Liu X., Xie M., Nie S., Chen Y., Xie J., Yu Q. (2019). Structural characteristics and functional properties of soluble dietary fiber from defatted rice bran obtained through *Trichoderma viride* fermentation. Food Hydrocoll..

[25] Moya P., Barrera V., Cipollone J., Bedoya C., Kohan L., Tolerdo A., Sisterna M. (2020). New isolates of *Trichoderma* spp. as biocontrol and plant growth-promoting agents in the pathosystem *Pyrenophora teres*-barley in Argentina. Biol. Control.

[26] Tomah A.A., Zhang Z., Alamer I.S.A., Khattak A.A., Ahmed T., Hu M., Wang D., Xu L., Li B., Wang Y. (2023). The potential of *Trichoderma*-mediated nanotechnology application in sustainable development scopes. Nanomaterials.

[27] Druzhinia I.S., Seidl-Seiboth V., Herrera-Estrella A., Horwitz B.A., Kenerley C.M., Monte E., Mukherjee P.K., Zeilinger S., Grigoriev I.V., Kubicek C.P. (2011). *Trichoderma*: The genomics of opportunistic success. Nat. Rev. Microbiol..

[28] Wakiyama M., Tanaka H., Yoshihara K., Hayashi S., Ohta K. (2008). Purification and properties of family-10 endo-1, 4-β-Xylanase from *Penicillium citrinum* and structural organization of encoding gene. J. Biosci. Bioeng..

[29] Lipsa R., Tudorachi N., Darie-Nita R.N., Oprica L., Vasile C., Chiriac A. (2016). Biodegradation of poly(lactic acid) and some of its based systems with *Trichoderma viride*. Int. J. Biol. Macromol..

[30] Peil S., Beckers S.J., Fischer J., Wurm F.R. (2020). Biodegradable, lignin-based encapsulation enables delivery of *Trichoderma reesei* with programmed enzymatic release against grapevine trunk diseases. Mat. Today Bio.

[31] Sun F.-S., Yu G.-H., Ning J.-Y., Zhu X.-D., Goodman B.A., Wu J. (2020). Biological removal of cadmiuum from biogas residues during vermicomposting, and the effect of earthworm hydrolysates on *Trichoderma guizhouense* sporulation. Biosour. Technol..

[32] Chinnapermal K., Govindasamy B., Paramasivam D., Dilipkumar A., Dhayalan A., Vadivel A., Sengodan K., Pachiappan P. (2018). Bio-pesticidal effects of *Trichoderma viride* formulated titanium dioxide nanoparticle and their physiological and biochemical changes on *Helicoverpa armigera* (Hub.). Pest Biochem. Physiol..

[33] Panchalingam H., Ashfield-Crook N., Naik V., Frenken R., Foster K., Tomlin R., Shapcott A., Kurtboke D.I. (2023). Testing the biocontrol ability of a *Trichoderma*-streptomycetes consortium against *Pyrrhoderma noxium* (Corner) L.W. Zhou and Y.C. Dai in soil. J. Fungi.

[34] Russo A., Pollastri S., Ruocco M., Monti M.M., Loreto F. (2022). Volatile organic compounds in the interaction between plants and beneficial microorganisms. J. Plant Interact..

[35] Tyskiewicz R., Nowak A., Ozimek E., Jaroszuk-Scisel J. (2022). *Trichoderma*: The current status of its application in agriculture for the biocontrol of fungal phytopathogens and stimulation of plant growth. Int. J. Mol. Sci..

[36] Arnold A.E., Mejia L.C., Kyllo D., Rojas E., Maynard Z., Robbins N., Herre E.A. (2003). Fungal endophytes limit pathogen damage in a tropical tree. Proc. Natl. Acad. Sci. USA.

[37] Redman R.S., Dunigan D.D., Rodriguez R.J. (2001). Fungal symbiosis: From mutualism to parasitism, who controls the outcome, host or invader?. New Phytol..

[38] Redman R.S., Sheehan K.B., Stout R.G., Rodriguez R.J., Henson J.M. (2002). Thermotolerance conferred to plant host and fungal endophyte during mutualistic symbiosis. Science.

[39] Waller F., Achatz B., Baltruschat H., Fodor J., Becker K., Fischer M., Heier T., Huckelhoven R., Neumann C., Von Wettstein D. (2005). The endophytic fungus *Piriformis indica* reprograms barley to salt-stress tolerance, disease resistance, and higher yield. Proc. Natl. Acad. Sci. USA.

[40] Marquez L.M., Redman R.S., Rodriguez R.J., Roossinck M.J. (2007). A virus in a fungus in a plant-three way symbioses required for thermal tolerance. Science.

[41] Allaga H., Zhumakayev A., Buchner R., Kocsube S., Szucz A., Vagvolgyi C., Kredics L., Hatvani L. (2021). Members of the *Trichoderma harzianum* species complex with mushroom pathogenic potential. Agronomy.

[42] Louw J.P., Korsten L. (2015). Pathogenicity and host susceptibility of *Penicillium* spp. on citrus. Plant Dis..

[43] Salinas M.C., Cavagnaro P.F. (2020). In vivo and in vitro screening for resistance against *Penicillium allii* in garlic accessions. Eur. J. Plant Pathol..

[44] Dugan F.M., Lupien S.L., Vahling-Armstrong C., Chastagner G.A., Schroeder B.K. (2017). Host range of *Penicillium* species causing blue mold of bulbs crops in Washington state and Idaho. Crop Prot..

[45] Zhu G., Wang X., Chen T., Wang S., Chen X., Song Z., Shi X., Laborda P. (2022). First report of *Aspergillus flavus* causing fruit rot on kiwifruit in China. Plant Dis..

[46] Poveda J., Eugui D., Abril-Urias P., Velasco P. (2021). Endophytic fungi as direct plant growth promoters for sustainable agricultural production. Symbiosis.

[47] Mathis K.A., Bronstein J.L. (2020). Our current understanding of commensalism. Annu. Rev. Ecol. Syst..

[48] Duan B., Gao Z., Reymick O.O., Ouyang Q., Chen Y., Long C., Yang B., Tao N. (2021). Cinnamaldehyde promotes the defense response in postharvest citrus fruit inoculated with *Penicillium digitatum* and *Geotrichum citri-aurantii*. Pest Biochem. Physiol..

[49] Liu K., Wang L., Jiang B., An J., Nian B., Wang D., Chen L., Ma Y., Wang X., Fan J. (2021). Effect of inoculation with *Penicillium chrysogenum* on chemical components and fungal communities in fermentation of Pu-erh tea. Food Res. Int..

[50] Fierro F., Vaca I., Castillo N.I., Garcia-Rico R.O., Chavez R. (2022). *Penicillium chrysogenum*, a vintage model with a cutting-edge profile in biotechnology. Microorganisms.

[51] Qi B., Jia F., Luo Y., Ding N., Li S., Shi F., Hai Y., Wang L., Zhu Z.-X., Liu X. (2022). Two new diterpenoids from *penicillium chrysogenum* MT-12, and endophytic fungus isolated from *Huperzia serrata*. Nat. Prod. Res..

[52] Newaz A.W., Yong K., Yi W., Wu B., Zhang Z. (2023). Antimicrobial metabolites from the Indonesian mangrove sediment-derived fungus *Penicillium chrysogenum* sp. ZZ1151. Nat. Prod. Res..

[53] Yang S., Zhang J., Liu Y., Feng W. (2023). Biodegradation of hydrocarbons by *Purpureocillium lilacinum* and *Penicillium chrysogenum* from heavy oil sludge and their potential for bioremediation of contaminated soil. Int. Biodeter. Biodegrade.

[54] Leitao A.L. (2009). Potential of *Penicillium* species in the bioremediation field. Int. J. Environ. Res. Public Health.

[55] Visagie C.M., Houbraken J., Frisvad J.C., Hong S.-B., Klaassen C.H.W., Perrone G., Seifert K.A., Varga J., Yaguchi T., Samson R.A. (2014). Identification and nomenclature of the genus *Penicillium*. Stud. Mycol..

[56] Hossain M.M., Sultana F., Kubota M., Hyakumachi M. (2008). Differential inducible defense mechanisms against bacterial speck pathogen in *Arabidopsis thaliana* by plant-growth-promoting-fungus *Penicillium* sp. GP16-2 and its cell free filtrate. Plant Soil.

[57] Ali S., Khan A.L., Ali L., Rizvi T.S., Khan S.A., Hussain J., Hamayun M., Al-Harrasi A. (2017). Enzyme inhibitory metabolites from endophytic *Penicillium citrinum* isolated from Boswellia sacra. Arch. Microbiol..

[58] Ali T., Inagaki M., Chai H., Wieboldt T., Rapplye C., Rakotondraibe L.H. (2017). Halogenated compounds from directed fermentation of *Penicillium concentricum*, an endophytic fungus of the Liverwort *Trichocolea tomentella*. J. Nat. Prod..

[59] Attia E.Z., Khalifa B.A., Shaban G.M., Abdelraheem W.M., Mustafa M., Abdelmohsen U.R., El-Katatny M.H. (2022). Discovering the chemical profile antimicrobial and antibiofilm potentials of the endophytic fungus *Penicillium chrysogenum* isolated from *Artemisia judaica* L. assisted with docking studies. S. Afr. J. Bot..

[60] Fu S.F., Wei J.Y., Chen H.W., Liu Y.Y., Lu H.Y., Chou J.Y. (2015). Indole-3-acetic acid: A widespread physiological code in interactions of fungi with other organisms. Plant Signal Behav..

[61] Shi Y., Xie H., Cao L., Zhang R., Xu Z., Wang Z., Deng Z. (2017). Effects of Cd- and Pb-resistant endophytic fungi on growth and phytoextraction of *Brassica napus* in metal-contaminated soils. Environ. Sci. Pollut. Res. Int..

[62] Mehmannavaz M., Nickavar B. (2022). Biotransformation of testosterone by the filamentous fungus *Penicillium pinophilum*. Arch. Microbiol..

[63] Tian H., Ma Y.J., Li W.Y., Wang J.W. (2018). Efficient degradation of triclosan by an endophytic fungus *Penicillium oxalicum* B4. Environ. Sci. Pollut. Res. Int..

[64] Yassin M.T., Mostafa A.A.-F., Al-Askar A.A., Sayed S.R.M., Rady A.M. (2021). Antagonistic activity of *Trichoderma harzianum* and *Trichoderma viride* strains against some fusarial pathogens causing stalk rot disease of maize, *in vitro*. J. King Saud. Univ. Sci..

[65] Gao S.S., Li X.M., Du F.Y., Li C.S., Proksch P., Wang B.G. (2010). Secondary metabolites from a marine-derived endophytic fungus *Penicillium chrysogenum* QEN-24S. Mar. Drugs.

[66] Gao S.S., Li X.M., Li C.S., Proksch P., Wang B.G. (2011). Penicisteroids A and B, antifungal and cytotoxic polyoxygenated steroids from the marine alga-derived endophytic fungus *Penicillium chrysogenum* QEN-24S. Bioorg. Med. Chem. Lett..

[67] Gao S.S., Li C.M., Zhang Y., Li C.S., Wang B.G. (2011). Conidiogenones H and I, two new diterpenes of Cyclopiane class from a marine-derived endophytic fungus Penicillium chrysogenum QEN-24S. Chem. Biodivers..

[68] Baitharu I., Jain V., Deep S.N., Shroff S., Sahu J.K., Naik P.K., Ilavazhagan G. (2014). Withanolide A prevents neurodegeneration by modulating hippocampal glutathione biosynthesis during hypoxia. PLoS ONE.

[69] Wang W., Zhai Y., Cao K., Tan H., Zhang R. (2016). Endophytic bacterial and fungal microbiota in sprouts, roots and stems of rice (*Oryza sativa* L.). Microbiol. Res..

[70] Wang W.G., Li A., Yan B.C., Niu S.B., Tang J.W., Li X.N., Du X., Challis G.L., Che Y., Sun H.D. (2016). LC-MS-guided isolation of Penicilfuranone A: A new antifibrotic furancarboxylic acid from the plant endophytic fungus *Penicillium* sp. sh18. J. Nat. Prod..

[71] Colovic M.B., Krstic D.Z., Lazarevic-Pasti T.D., Bondzic A.M., Vasic V.M. (2013). Acetylcholinesterase inhibitors: Pharmacology and toxicology. Curr. Neuropharmacol..

[72] Ateba J.E.T., Toghueo R.M.K., Awantu A.F., Mba’ning B.M., Gohlke S., Sahal D., Rodrigues-Filho E., Tsamo E., Boyom F.F., Sewald N. (2018). Antiplasmodial properties and cytotoxicity of endophytic fungi from *Symphonia globulifera* (Clusiaceae). J. Fungi.

[73] Toghueo R.M.K., Boyom F.F. (2020). Endophytic *Penicillium* species and their agricultural, biotechnological, and pharmaceutical applications. 3 Biotech.

[74] Leitao A.L., Garcia-Estrada C., Ullan R.V., Guedes S.F., Martin-Jimenez P., Mendes B., Martin J.F. (2012). *Penicillium chrysogenum* var. halophenolicum, a new halotolerant strain with potential in the remediation of aromatic compounds in high salt environments. Microbiol. Res..

[75] Ferreira-Guedes S., Leitao A.L. (2018). Simultaneous removal of dihydroxybenzenes and toxicity reduction by *Penicillium chrysogenum* var. halophenolicum under saline conditions. Ecotoxicol. Environ. Saf..

[76] Francis F., Jaber K., Colinet F., Portetelle D., Haubruge E. (2011). Purification of a new fungal mannose-specific lectin from *Penicillium chrysogenum* and its aphicidal properties. Fungal Biol..

[77] Garcia-Estrada C., Martin J.F., Cueto L., Barreiro C. (2020). Omics approaches applied to *Penicillium chrysogenum* and Penicillin production: Revealing the secrets of improved productivity. Genes.

[78] Martin J.F. (2020). Insight into the genome of diverse *Penicillium chrysogenum* strains: Specific genes, cluster duplications and DNA fragment translocatins. Int. J. Mol. Sci..

[79] Sonderegger C., Galgoczy L., Garrigues S., Fizil A., Borics A., Manzanares P., Hegedus N., Huber A., Marcos J.F., Batta G. (2016). A *Pencillium chrysogenum*-based expression system for the production of mall, cysteine-rich antifungal proteins for structural and functional analyses. Microb. Cell Fact..

[80] Estrada-Rivera M., Hernandez-Onate M.A., Dautt-Castro M., Gallardo-Negrete J.D.J., Rebolledo-Prudencio O.G., Uresti-Rivera E.E., Arenas-Huertero C., Herrera-Estrella A., Casas-Flores S. (2020). IPA-1 a putative chromatin remodeler/helicase-related protein of *Trichodermis virens* plays important roles in antibiosis against *Rhizoctonia solani* and induction of *Arabidopsis* systemic disease resistance. Mol. Plant Microbe Interact..

[81] Garcia-Rico R.O., Martin J.F., Fierro F. (2011). Heterotrimeric Ga protein Pga1 from *Penicillium chrysogenum* triggers germination in response to carbon sources and affects negatively resistance to different stress conditions. Fungal Gen. Biol..

[82] Akaniro I.R., Chibuikde I.V., Onwujekwe E.C., Gbadamosti F.A., Enyi D.O., Onwe O.N. (2023). *Penicillium* species as chassis for biomanufacturing and environmental sustainability in the modern era: Progress, challenges, and future perspective. Fungal. Biol. Rev..

[83] Meng L., Sun P., Tang H., Li L., Draeger S., Schulz B., Krohn K., Hussain H., Zhang W., Yi Y. (2011). Endophytic fungus *Penicillium chrysogenum*, a new source of hypocrellins. Biochem. Syst. Ecol..

[84] Qin J., Teng J., Li Z., Xia N., Wei B., Huang L. (2022). Expression of citrinin biosynthesis gene in Liupao tea and effect of *Penicillium citrinum* on tea quality. Fungal Gene Biol..

[85] Guijarro B., Larena I., Melgarejo P., Cal A.D. (2017). Adaptive conditions and safety of the application of *Penicillium frequentans* as a biocontrol agent on stone fruit. Int. J. Food Microbiol..

[86] Arunthirumeni M., Vinitha G., Shivakumar M.S. (2023). Antifeedant and larvicidal activity of bioactive compounds isolated from entomopathogenic fungi *Penicillium* sp. for the control of agricultural and medically important insect pest (*Spodoptera litura* and *Culex quinquefasciatus*). Parasitol. Int..

[87] Karpova N.V., Yaderets V.V., Glagoleva E.V., Petrova K.S., Ovchinnikov A.I., Dzhavakhiya V.V. (2021). Antifungal activity of the dry biomass of *Penicillium chrysogenum* F-24-28 and Is application in combination with azoxystrobin for efficient crop protection. Agriculture.

[88] Sikandar A., Zhang M., Wang Y., Zhu X., Liu X., Fan H., Xuan Y., Chen L., Duan Y. (2020). In vitro evaluation of *Penicillium chrysogenum* Snef1216 against *Meloidogyne incognita* (root-knot nematode). Sci. Rep..

[89] Khan S.A., Hamayun M., Yoon H., Kim H.-Y., Suh S.-J., Hwang S.-K., Kim J.-M., Lee I.-J., Choo Y.-S., Yoon U.-H. (2008). Plant growth promotion and *Penicillium citrinum*. BMC Microbiol..

[90] Nguyen H.C., Lin K.-H., Nguyen T.P., Le H.S., Ngo K.N., Pham D.C., Tran T.N., Su C.-H., Barrow C.J. (2023). Isolation and cultivation of *Penicillium citrinum* for biological control of *Spodoptera litura* and *Plutella xylostella*. Fermentation.

[91] Babu J.V., Popay A.J., Miles C.O., Wilkins A.L., di Menna M.E., Finch S.C. (2018). Identification and structure elucidation of Janthitrems A and D from *Penicillium janthinellum* and determination of tremorgenic and anti-insect activity of Janthitrems A and B. J. Agric. Food Chem..

[92] Li J., Yang X., Lin Y., Yuan J., Lu Y., Zhu X., Li J., Li M., Lin Y., He J. (2014). Meroterpenes and azaphilones from marine mangrove endophytic fungus *Penicillium* 303#. Fitoterapa.

[93] Li X.D., Miao F.P., Liang X.R., Ji N.Y. (2014). Meroterpenes from an algicolous strain of *Penicillium echinulatum*. Magn. Reson. Chem..

[94] Katoch M., Pull S. (2017). Endophytic fungi associated with *Monarda citriodora*, an aromatic and medicinal plant and their biocontrol potential. Pharm. Biol..

[95] Li Z.F., Wang L.F., Feng Z.L., Zhao L.H., Shi Y.Q., Zhu H.Q. (2014). Diversity of endophytic fungi from different *Verticillium*-wilt-resistant *Gossypium hirsutum* and evaluation of antifungal activity against *Verticillium dahliae* in vitro. J. Microbiol. Biotechnol..

[96] Hassan S.E. (2017). Plant growth-promoting activities for bacterial and fungal endophytes isolated from medicinal plant of *Teucrium polium* L. J. Adv. Res..

[97] Ownley B.H., Benson D.M. (1992). Evaluation of *Penicillium janthinellum* as a biological control of phytophthora root rot of Azalea. J. Amer. Soc. Hort. Sci..

[98] Gu K., Chen C.-Y., Selvaraj P., Pavagadhi S., Yeap Y.T., Swarup S., Zheng W., Naqvi N.I. (2023). *Penicillium citrinum* provides transkingdom growth benefits in Choy Sum (*Brassica rapa* var. parachinensis). J. Fungi.

[99] Dong H., Zhang X., Choen Y., Zhou Y., Li W., Li Z. (2006). Dry mycelium of *Penicillium chrysogenum* protects cotton plants against wilt diseases and increases yield under field conditions. Crop Prot..

[100] Sikandar A., Zhang M.Y., Zhu X.F., Wang Y.Y., Ahmed M., Iqbal M.F., Javeed A., Xuan Y.H., Fan H.Y., Liu X.Y. (2019). Efficacy of *Penicillium chrysogenum* strain SNEF1216 against root-knot nematodes (*Meloidogyne incognita*) in cucumber (*Cucumis sativus* L.) under greenhouse conditions. Appl. Ecol. Environ. Res..

[101] Li S.-Y., Yang X.-Q., Chen J.-X., Wu Y.-M., Yang Y.-B., Ding Z.-T. (2022). The induced cryptic metabolites and antifungal activities from culture of *Penicillium chrysogenum* by supplementing with host *Ziziphus jujuba* extract. Phytochemistry.

[102] Galeano R.M.S., Silva S.M., Yonekawa M.K.A., Guimaraes N.C.D.A., Giannesi G.C., Masui D.C., Correa B.O., Brasil M.D.S., Zanoelo F.F. (2023). *Penicillium chrysogenum* strain 34-P promotes plant growth and improves initial development of maize under saline conditions. Rhizosphere.

[103] Murali M., Sudisha J., Amruthesh K.N., Ito S.-I., Shetty H.S. (2013). Rhizosphere fungus *Penicillium chrysogenum* promotes growth and induces defence-related genes and downy mildew disease resistance in pearl millet. Plant Biol..

[104] Gotlieb D., Oka Y., Ben-Daniel B.-H., Cohen Y. (2003). Dry mycelium of *Penicillium chrysogenum* protects cucumber and tomato plants against root-knot nematode *Meloidogyne javanica*. Phytoparasitica.

[105] Khan A.L., Waqas M., Khan A.R., Hussain J., Kang S.-M., Gilani S.A., Hamayun M., Shin J.-H., Kamran M., Al-Harrasi A. (2013). Fungal endophyte *Penicillium janthinellum* LK5 improves growth of ABA-deficient tomato under salinity. World J. Microbiol. Biotechnol..

[106] Khan A.L., Waqas M., Hussain J., Al-Harrasi A., Hamayun M., Lee I.-J. (2015). Phytohormones enabled endophytic fungal symbiosis improve aluminum phytoextraction in tolerant *Solanum lycopersicum*: A examples of *Penicillium janthinellum* LK5 and comparison with exogenous GA_3_. J. Hazard. Mat..

[107] Ting A.S.Y., Mah S.W., Tee C.S. (2012). Evaluating the feasibility of induced host resistance by endophytic isolate *Penicillium citrinum* BTF08 as a control mechanism for *Fusarium* wilt in banana plantlets. Biol. Control.

[108] Chen S., Dong H., Fan Y., Li W., Cohen Y. (2006). Dry mycelium of *Penicillium chrysogenum* induces expression of pathogenesis-related protein genes and resistance against wilt diseases in Bt transgenic cotton. Biol. Control.

[109] Dong H., Li W., Zhang D., Tang W. (2003). Differential expression of induced resistance by an aqueous extract of killed *Penicillium chrysogenum* against Verticillium wilt of cotton. Crop Prot..

[110] Wang H., Mo S., Xia Q., Zhao Z., Chen X., Shen X., Yin C., Mao Z. (2022). The interaction of the pathogen *Fusarium proliferatum* with *Trichoderma asperellum* characterized by transcriptome changes in apple rootstock roots. Physiol. Mol. Plant Pathol..

[111] Li X., Leng J., Yu L., Bai H., Li X., Wisniewski M., Liu J., Sui Y. (2022). Efficacy of the biocontrol agent *Trichoderma hamatum* against *Lasiodiplodia theobromae* on macadamia. Front. Microbiol..

[112] Ibiang S.R., Usami T., Sakamoto K., Ibiang Y.B. (2023). Lettuce tolerance to verticillium wilt after inoculation with *Penicillium pinophilum* and *Rhizophagus intraradices*. Physiol. Mol. Plant Pathol..

[113] Dong H., Cohen Y. (2001). Extracts of killed *Penicillium chrysogenum* induce resistance against *Fusarium* wilt of melon. Phytoparasitica.

[114] Shen F., Wang G., Liu X., Zhu S. (2023). Exogenous inoculation of endophyte *Penicillium* sp. alleviated pineapple internal browning during storage. Heliyon.

[115] Maity A., Pal R.K., Chandra R., Singh N.V. (2014). *Penicillium pinophilum*—A novel microorganism for nutrient management in pomegranate (*Punica granatum* L.). Sci. Hortic..

[116] Rosa C.A.R., Keller K.M., Oliveira A.A., Almeida T.X., Keller L.A.M., Marassi A.C., Kruger C.D., Deveza M.V., Monteiro B.S., Nunes L.M.T. (2010). Production of citreoviridin by *Penicillium citreonigrum* strains associated with rice consumption and beriberi cases in the Maranhao state, Brazil. Food Additt. Contam. Part A Chem. Anal. Control Expo. Risk Assess.

[117] Yang X.C., Duan Y.M., Wang X.X., Wang J.G., Xu X.Y., Chen S.L., Yin P., Zhu S., Li X.J., Chen S.Y. (2013). The effect of dry mycelium of *Penicillium chrysogenum* on the growth of flue-cured tobacco on floating system and resistance against tobacco mosaic under field conditions. J. Yunnan Agric. Univ..

[118] Zhong Y., Li Y., Huang K., Chen Z.-Z., Fu J., Liu C.-M., Chen S.-Y., Wang J.-G. (2021). Crude peptides extracted from dry mycelium of *Penicillium chrysogenu* serve as a micro-associated molecular pattern to induce systemic resistance against tobacco mosaic virus in tobacco. Physiol. Mol. Plant Pathol..

[119] Zhong Y., Li Y., Chen Z., Fu J., Li X., Zhang B., Chen S., Wang J. (2021). Treatment of *Penicillium chrysogenum* extracts (PDMP) restricts the spread of *Tobacco mosaic* virus by priming callose deposition in *Nicotiana benthamiana*. Physiol. Mol. Plant Pathol..

[120] Zhong Y., Pen J.-J., Chen Z.-Z., Xie H., Luo D., Dai J.-R., Yan F., Wang J.-G., Dong H.-Z., Chen S.-Y. (2015). Dry mycelium of *Penicillium chrysogenum* activates defense responses and restricts the spread of *Tobacco Mosaic Virus* in tobacco. Physiol. Mol. Plant Pathol..

[121] Wang J., Chen S. (2012). Dry mycelium of *Penicillium chrysogenum* protect flue-cured tobacco against brown spot and wildfire disease. New Biotechnol..

[122] Li Y., Jiao M., Li Y., Zhong Y., Li X., Chen Z., Chen S., Wang J. (2021). *Penicillium chrysogenum* polypeptide extract protects tobacco plants from tobacco mosaic virus infection through modulation of ABA biosynthesis and callose priming. J. Experim. Bot..

[123] Fu J., Zhang S., Wu J., Chen Y., Zhong Y., Zhou Y., Wang J., Chen S. (2020). Structural characterization of a polysaccharide from dry mycelium of *Penicillium chrysogenum* that induces resistance to Tobacco mosaic virus in tobacco plants. Int. J. Biol. Macromol..

[124] Kaur R., Saxena S. (2023). *Penicillium citrinum*, a drought-tolerant endophytic fungus isolated from wheat (*Triticum aestivum* L.) leaves with plant growth-promoting abilities. Curr. Microbiol..

[125] Narwade J.D., Odaneth A.A., Lele S.S. (2023). Solid-state fermentation in an earthen vessel: *Trichoderma viride* spore-based biopesticide production using corn cobs. Fungal Biol..

[126] Juric S., Dermic E., Topolovec-Pintaris S., Bedek M., Vincekovic M. (2019). Physicochemical properties and release characteristics of caldium alginate microspheres loaded with *Trichoderma viride* spores. J. Integr. Agric..

[127] Nabilah B., Purnomo A.S., Prasetyoko D., Rohmah A.A. (2023). Methylene blue biodecolotization and biodegradation by immobilized mixed cultures of *Trichoderma viride* and *Ralstonia pickettii* into SA-PVA-Bentionite matrix. Arab J. Chem..

[128] Xu S., Luo Y., Han Z., Zhang T., Sun L., Zheng G., Wang K., Cheng Z. (2023). Diatomite-*Trichoderma viride* composite microspheres for selective removal of anionic dyes and copper ions. J. Water Process Eng..

[129] Alonso-Ramirez A., Poveda J., Martin H., Hermosa R., Monte E., Nicolas C. (2014). Salicylic acid prevents *Trichoderma harzianum* from entering the vascular system of roots. Mol. Plant Pathol..

[130] Taylor J.T., Harting R., Shalaby S., Kenerley C.M., Braus G.H., Horwitz B.A. (2022). Adhesion as a focus in *Trichoderma*-root interactions. J. Fungi.

[131] Pfordt A., Gaumann P., von Tiedemann A. (2023). Pathogenicity of *Trichoderma afroharzianum* in cereal crops. Pathogen.

[132] Pfordt A., Schiwek S., Karlovsky P., von Tiedemann A. (2020). *Trichoderma afroharzianum* ear rot- A new disease on maize in Europe. Front. Agron..

[133] Sanna M., Pugliese M., Gullino M.L., Mezzalama M. (2022). First report of *Trichoderma afroharzianum* causing seed rot on maize in Italy. Plant Diseases.

[134] Schmoll M., Esquivel-Naranjo E.U., Herrera-Estrella A. (2010). Trichoderma in the light of day-Physiology and development. Fungal Genet. Biol..

[135] Kubicek C.P., Herrera-Estrella A., Seidl-Seiboth V., Martinez D.A., Druzhini I.S., Thon M., Zeilinger S., Casas-Flores S., Horwitz B.A., Mukherjee P.K. (2011). Comparative genome sequence analysis underscores mycoparasitism as the ancestral life style of *Trichoderma*. Genome Biol..

[136] Schuster A., Bruno K.S., Collett J.R., Baker S.E., Seiboth B., Kubicek C.P., Schmoll M. (2012). A versatile toolkit for high throughput functional genomics with *Trichoderma reesei*. Biotechnol. Biofuels.

[137] Chammem H., Nesler A., Pertot I. (2021). Wood pellets as carriers of conidia of *Trichoderma atroviride* SC1 for soil application. Fungal Biol..

[138] Zhang G.-Z., Yang H.-T., Zhang X.-J., Zhou F.-Y., Wu X.-Q., Xie X.-Y., Zhao X.-Y., Zhou H.-Z. (2022). Five new species of *Trichoderma* from moist soils in China. Mycokeys.

[139] Woo S.L., Hermosa R., Lorito M., Monte E. (2023). *Trichoderma*: A multipurpose, plant-beneficial microorganism for eco-sustainable agriculture. Nat. Rev. Microbiol..

[140] Verma H., Kumar D., Kumar V., Kumari M., Singh S.K., Sharma V.K., Droby S., Santoyo G., White J.F., Kumar A. (2021). The potential application of endophytes in management of stress from drought and salinity in crop plants. Microorganisms.

[141] Perera D.S., Tharaka W.G.H., Amarasinghe D., Wickramarachchi S.R. (2023). Extracellular extracts of antagonistic fungi, *Trichoderma longibrachiatum* and *Trichoderma viride*, as larvicides against dengue vectors, *Aedes aegypti* and *Aedes albopictus*. Acta Trop.

[142] Moussa Z., Alanazi Y.F., Khateb A.M., Eldadamony N.M., Ismail M.M., Saber W.I., Darwish D.B.E. (2023). Domiciliation of *Trichoderma asperellum* suppresses *Globiosporangium ultimum* and promotes pea growth, ultrastructure, and metabolic features. Microorganisms.

[143] Fayzalla E., Sadik E., Elwakil M., Gomah A. (1994). Soil solarization for controlling *Cephalosporium maydis*, the cause of late wilt disease of maize in Egypt. Egypt J. Phytopathol..

[144] Legein M., Smets W., Vandenheuvel D., Eilers T., Muyshondt B., Prinsen E., Samson R., Lebeer S. (2020). Modes of action of microbial biocontrol in the phyllosphere. Front. Microbiol..

[145] Alghuthaymi M.A., Abd-Elsalam K.A., AboDalam H.M., Ahmed F.K., Ravichandran M., Kalia A., Rai M. (2022). *Trichoderma*: An eco-friendly source of nanomaterials for sustainable agroecosystems. J. Fungi.

[146] Zhang F., Xu X., Huo Y., Xiao Y. (2019). Trichoderma-inoculation and mowing synergistically altered soil available nutrients, rhizosphere chemical compounds and soil microbial community, potentially driving alfalfa growth. Front. Microbiol..

[147] Molla A.H., Manjurul Haque M., Amdadul Haque M., Ilias G.N.M. (2012). *Trichoderma*-enriched biofertilizer enhances production and nutritional quality of tomato (*Lycopersicon esculentum* Mill.) and minimizes NPK fertilizer use. Agric. Res..

[148] Sun J., Yuan X., Li Y., Wang X., Chen J. (2019). The pathway of 2,2-dichlorovinyl dimethyl phosphate (DDVP) degradation by *Trichoderma atroviride* strain T23 and characterization of a paraoxonase-like enzyme. Appl. Microbiol. Biotechnol..

[149] Lodi R.S., Peng C., Dong X., Deng P., Peng L. (2023). *Trichoderma hamatum* and its benefits. J. Fungi.

[150] Velasco P., Rodriguez V.M., Soengas P., Pveda J. (2021). Content and antioxidant potential of different leafy *Brassica* vegetables. Plants.

[151] Krause M.S., De Ceuster T.J.J., Tiquia S.M., Michel F.C., Madden L.V., Hoitink H.A.J. (2003). Isolation and characterization of rhizobacteria from composts that suppress the severity of bacterial leaf spot of radish. Phytopathology.

[152] Daba A., Berecha G., Tadesse M., Belay A. (2021). Evaluation of the herbicidal potential of some fungal species against *Bidens Pilosa*, the coffee farming wees. Saudi. J. Biol. Sci..

[153] Wen C., Xiong H., Wen J., Wen X., Wang C. (2020). Trichoderma species attract *Coptotermes formosanus* and antagonize termite pathogen *Metarhizium anisopliae*. Front. Microbiol..

[154] Lana M., Simon O., Velasco P., Rodriguez V.M., Caballero P., Poveda J. (2023). First study on the root endophytic fungus *Trichoderma hamatum* as an entomopathogen: Development of a fungal bioinsecticide against cotton leafworm (*Spodoptera littoralis*). Microbiol. Res..

[155] Rai S., Kashyap P.L., Kumar S., Srivastava A.K., Ramteke P.W. (2016). Identification, characterization and phylogenetic analysis of antifungal *Trichoderma* from tomato rhizosphere. Springerplus.

[156] Sood M., Kapoor D., Kumar V., Sheteiwy M.S., Ramakrishnan M., Landi M., Araniti F., Sharma A. (2020). *Trichoderma*: The secrets of a multitalented biocontrol agent. Plants.

[157] Moran-Diez E., Hermosa R., Ambrosino P., Cardoza R.E., Gutierrez S., Lorito M., Monte E. (2009). ThPG1 endopolygalacturonase is required for the *Trichoderma harzianum*-plant beneficial interaction. Mol. Plant Microbe Interact..

[158] Jaroszuk-Scisel J., Tyskiewicz R., Nowak A., Ozimek E., Majewska M., Hanaka A., Tyskiewicz K., Pawlik A., Janusz G. (2019). Phytohormones (auxin, gibberellin) and ACC deaminase in vitro synthesized by the mycoparasitic *Trichoderma* DEMTkZ3A0 strain and changes in the level of auxin and plant resistance markers in wheat seedlings inoculated with this strain conidia. Int. J. Mol. Sci..

[159] An X.-Y., Cheng G.-H., Gao H.-X., Li X.-F., Yang Y., Li D., Li Y. (2022). Phylogenetic analysis of *Trichoderma* species associated with green mold disease on mushrooms and two new pathogens on *Ganoderma sichuanense*. J. Fungi.

[160] Krause M.S., Madden L.V., Hoitink H.A.J. (2001). Effect of potting mix microbial carrying capacity on biological control of *Rhizoctonia* damping-off of radish and *Rhizoctonia* crown root rot of poinsettia. Phytopathology.

[161] Alfano G., Lewis Ivey M.L., Cakir C., Bos J.I.B., Miller S.A., Madden L.V., Kamoun S., Hoitink H.A.J. (2007). Systemic modulation of gene expression in tomato by *Trichoderma hamatum* 382. Phytopathology.

[162] Baazeem A., Almanea A., Manikandan P., Alorabi M., Vijayaraghavan P., Abdel-Hadi A. (2021). In vitro antibacterial, antifungal, nematocidal and growth promoting activities of *Trichoderma hamatum* fb10 and its secondary metabolites. J. Fungi.

[163] Wamani A.O., Muthomi J.W., Mutitu E., Waceke W.J. (2023). Efficacy of microbial antagonists in the management of bacterial wilt of field-grown tomato. J. Nat. Pestic. Res..

[164] Shaw S., Le Cocq K., Paszkiewicz K., Moore K., Winsbury R., De Torres Zabala M., Studholme D.J., Salmon D., Thornton C.R., Grant M.R. (2016). Transcriptional reprogramming underpins enhanced plant growth promotion by the biocontrol fungus *Trichoderma hamatum* gd12 during antagonistic interactions with *Sclerotinia sclerotiorum* in soil. Mol. Plant Pathol..

[165] Studholme D.J., Harris B., Le Cocq K., Winsbury R., Perera V., Ryder L., Ward J.L., Beale M.H., Thornton C.R., Grant M. (2013). Investigating the beneficial traits of *Trichoderma hamatum* GD12 for sustainable agriculture-insights from genomics. Front. Plant Sci..

[166] Sun W., Shahrajabian M.H. (2024). Survey on nitrogenase evolution by considering the importance of nitrogenase, its structure, and mechanism of nitrogenase. Not. Bot. Horti Agrobot. Cluj-Napoca.

[167] Sun W., Shahrajabian M.H., Kuang Y., Wang N. (2024). Amino acids biostimulants and protein hydrolysates in agricultural sciences. Plants.

[168] Sun W., Shahrajabian M.H., Soleymani A. (2024). The roles of plant-growth-promoting rhizobacteria (PGPR)-based biostimulants for agricultural production systems. Plants.

[169] Wang L., Zhang Y., Wang Y., Suo M., Wu H., Zhao M., Yang H. (2022). Inoculation with *Penicillium citrinum* aids ginseng in resisting *Fusarium oxysporum* by regulating the root and rhizosphere microbial communities. Rhizosphere.

[170] Liu R., Chen M., Gao J., Luo M., Wang G. (2023). Identification of antagonistic fungi and their antifungal activities against aconite root rot pathogens. Plant Signal Behav..

[171] Poveda J., Rodriguez V.M., Abilleira R., Velasco P. (2023). *Trichoderma hamatum* can act as inter-plant communicator of foliar pathogen infections by colonizing the roots of nearby plants: A new inter-plant wired communication. Plant Sci..

[172] Kottb M., Gigolashvili T., GroBkinsky D.K., Piechulla B. (2015). *Trichoderma* volatiles effecting *Arabidopsis*: From inhibition to protection against phytopathogenic fungi. Front. Microbiol..

[173] Garnica-Vergara A., Barrera-Ortiz S., Munoz-Parra E., Raya-Gonzalez J., Mendez-Bravo A., Macias-Rodriguez L., Ruiz-Herrera L.F., Lopez-Bucio J. (2016). The volatile 6-pentyl-2H-pyran-2-one from *Trichoderma atroviride* regulates *Arabidopsis thaliana* root morphogenesis via auxin signaling and ETHYLENE INSENSITIVE 2 functioning. New Phytol..

[174] Boukaew S., Kumla J., Prasertsan P., Cheirsilp B., Petlamul W., Srinuanpan S. (2023). In vitro and in situ antifungal properties of a *Trichoderma asperelloides* SKRU-01 against aflatoxigenic *aspergillus* species. Food Control..

[175] Haouhach S., Karkachi N., Oguiba B., Sisaoui A., Chamorro I., Kihal M., Monte E. (2020). Three new reports of *Trichoderna* in Algeria: T. atrobrunneum (South), *T. longibrachiatum* (South), and *T. afroharzianum* (Northwest). Microorganisms.

[176] Ma J., Tsegaye E., Li M., Wu B., Jiang X. (2020). Biodiversity of *Trichoderma* from grassland and forest ecosystems in Northern Xinjiang, China. 3 Biotech.

[177] Saravanakumar K., Fan L., Fu K., Yu C., Wang M. (2016). Cellulase from *Trichoderma harzianum* interacts with roots and triggers induced systemic resistance to foliar disease in maize. Sci. Rep..

[178] Inglis P.W., Mello S.C.M., Martins I., Silva J.B.T., Macedo K., Sifuentes D.N., Valadares-Inglis M.C. (2020). *Trichoderma* from Brazilian garlic and onion crop soils and description of two new species: *Trichoderma azevedoi* and *Trichoderma peberdyi*. PLoS ONE.

[179] Jiang Y., Wang J.L., Chen J., Mao L.J., Feng X.X., Zhang C.L., Lin F.C. (2016). *Trichoderma* biodiversity of agricultural fields in east China reveals a gradient distribution of species. PLoS ONE.

[180] Cummings N., Ambrose A., Braithwaite M., Bissett J., Roslan H.A., Abdullah J., Stewart A., Agbayani F.V., Steyaert J., Hill R.A. (2016). Diversity of root-endophytic *Trichoderma* from Malaysian Borneo. Mycol. Progr..

[181] Swain H., Adak T., Mkherjee A.K., Sarangi S., Samal P., Khandual A., Jena R., Bhattacharyya P., Naik S.K., Mehetre S.T. (2021). Seed biopriming with *Trichoderma* stains isolated from tree bark improves plant growth, antioxidative defense system in rice and enhance straw degradation capacity. Front. Microbiol..

[182] Blaszczyk L., Strakowska J., Chelkowski J., Gabka-Buszek A., Kaczmarek J. (2016). *Trichoderma* species occurring on wood with decay symptoms in mountains forests in Central Europe: Genetic and enzymatic characterization. J. Appl. Genet..

[183] Bohacz J., Kornillowicz-Kowalska T. (2020). Modification of post-industrial lignin by fungal strains of the genus *Trichoderma* isolated from different composting stages. J. Environ. Manag..

[184] Kubiak A., Wolna-Maruwka A., Pilarska A.A., Niewiadomska A., Piotrowska-Cyplik A. (2023). Fungi of the *Trichoderma* genus: Future perspectives of benefits in sustainable agriculture. Appl. Sci..

[185] Ruocco M., Lanzuise S., Lombardi N., Woo S.L., Vinale F., Marra R., Varlese R., Manganiello G., Pascale A., Scala V. (2015). Multiple roles and effects of a novel *Trichoderma hydrophobin*. Mol. Plant-Microbe Interact..

[186] Ramirez-Valdespino C.A., Porras-Troncoso M.D., Corrales-Escobosa A.R., Wrobel K., Martinez-Hernandez P., Olmedo-Monfil V. (2018). Functional characterization of TvVyt2, a member of the p450 monoocygenases from *Trichoderma virens* relevant during the association with plants and mycoparasitism. Mol. Plant-Microbe Interact..

[187] Rimkus A., Namina A., Dzierkale M.T., Grigs O., Senkovs M., Larsson S. (2023). Impact of growth conditions on the viability of *Trichoderma asperellum* during storage. Microorganisms.

[188] Brotman Y., Briff E., Viterbo A., Chet I. (2008). Role of swollenin, an expansin-like protein from *Trichoderma*, in plant root colonization. Plant Physiol..

[189] Zhang S., Gan Y., Xu B. (2016). Application of plant-growth-promoting fungi *Trichoderma longibrachiatum* T6 enhances tolerance of wheat to salt stress through improvement of antioxidative defense system and gene expression. Front. Plant Sci..

[190] Ghorbanpour A., Salimi A., Ghanbary M.A.T., Pirdashti H., Dehestani A. (2018). The effect of *Trichoderma harzianum* in mitigating low temperature stress in tomato (*Solanum lycopersicum* L.) plants. Sci. Hortic..

[191] Gallou A., Cranenbrouk S., Declerck S. (2008). *Trichoderma harzianum* elicits defence response genes in roots of potato plantlets challenged by *Rhizoctonia solani*. Eur. J. Plant Pathol..

[192] Wang Y., Hou X., Deng J., Yao Z., Lyu M., Zhang R. (2020). Auxin response factor 1 acts as a positive regulator in the response of poplar to *Trichoderma asperellum* inoculation in over-expressing plants. Plants.

[193] Kumar V., Koul B., Taak P., Yadav D., Song M. (2023). Journey of *Trichoderma* from pilot scale to mass production: A review. Agriculture.

[194] Abbas A., Mubeen M., Zheng H., Sohail M.A., Shakeel Q., Solanki M.K., Iftikhar Y., Sharma S., Kashyap B.K., Hussain S. (2022). *Trichoderma* spp. Genes involved in the biocontrol activity against *Rhizoctonia solani*. Front. Microbiol..

[195] Marcello C.M., Steindorff A.S., da Silva S.P., do Nascimento Silva R., Bataus L.A.M., Ulhoa C.J. (2010). Expression analysis of the exo-B-1, 3-glucanase from the mycoparasitic fungus *Trichoderma asperellum*. Microbiol. Res..

[196] Mukherjee P.K., Latha J., Hadar R., Horwitz B.A. (2004). Role of two G-protein alpha subunits, TgaA and TgaB, in the antagonism of plant pathogens by *Trichoderma virens*. Appl. Environ. Microbiol..

[197] Moran-Diez M.E., Martinez de Alba A.E., Rubio M.B., Hermosa R., Monte E. (2021). *Trichoderma* and the plant heritage priming responses. J. Fungi.

[198] Vizcaino J., Cardoza R., Hauser M., Hermosa R., Rey M., Llobell A., Becker J., Gutierrez S., Monte E. (2006). ThPTR2, a di/tri-peptide transporter gene from *Trichoderma harzianum*. Fungal Genet. Biol..

[199] Tijerino A., Cardoza R.E., Moraga J., Malmierca M.G., Vicente F., Aleu J., Collado I.G., Gutierrez S., Monte E., Hermosa R. (2011). Overexpression of the trichodiene synthase gene trip5 increases *trichodermin* production and antimicrobial activity in *Trichoderma brevicompactum*. Fungal Genet. Biol..

[200] Dixit P., Mukherjee P.K., Ramachandran V., Eapen S. (2011). Glutathone transferase from *Trichoderma virens* enhances cadmium tolerance without enhancing its accumulation in transgenic *Nicotiana tabacum*. PLoS ONE.

[201] Zhong Y.H., Wang T.H., Wang X.L., Zhang G.T., Yu H.N. (2009). Identification and characterization of a novel gene, TrCCD1, and its possible function in hyphal growth and conidiospore development of *Trichoderma reesei*. Fungal Genet. Biol..

[202] Migheli Q. (2008). Biodiversity of the Genus *Trichoderma* and Identification of Marker Genes Involved in the Antagonism Between *Trichoderma* spp. and Plant Pathogenic Fungi. Ph.D. Thesis.

[203] Samolski I., Rincon A.M., Pinzon L.M., Viterbo A., Monte E. (2012). The qid74 gene from *Trichoderma harzianum* has a role in root architecture and plant biofertilization. Microbiology.

[204] Mukherjee M., Mukherjee P.K., Kale S.P. (2007). cAMP signaling is involved in growth, germination, mycoparasitism and secondary metabolism in *Trichoderma virens*. Microbiology.

[205] Min S.Y., Kim B.-G., Lee C., Hur H.-G., Ahn J.-H. (2002). Purification, characterization, and cDNA cloning of xylanase from fungus *Trichoderma* strain SY. J. Microbiol. Biotechnol..

[206] Guzman-Guzman P., Aleman-Duarte M.I., Delaye L., Herrera-Estrella A., Olmedo-Monfil V. (2017). Identification of effector-like proteins in *Trichoderma* spp. and role of a hydrophobin in the plant-fungus interaction and mycoparasitism. BMC Genet..

[207] Atanasova L., Gruber S., Lichius A., Radebner T., Abendstein L., Munsterkotter M., Stralis-Pavese N., Labaj P.P., Kreil D.P., Zeilinger S. (2018). The Gpr1-regulated Sur7 family protein Sfp2 is required for hyphal growth and cell wall stability in the mycoparasite *Trichoderma atroviride*. Sci. Rep..

[208] Guo R., Ji S., Wang Z., Zhang H., Wang Y., Liu Z. (2021). *Trichoderma asperellum* xylanases promote growth and induce resistance in poplar. Microbiol. Res..

[209] Nabi S.U., Raja W.H., Sharma A., Malik G. (2017). Evaluation of different substrates for development of *Trichoderma harzianum* based stock cultures and their utilization in management of chilli wilt disease. Chem. Sci. Rev. Lett..

[210] Mohiddin F., Bashir I., Padder S.A., Hamid B. (2017). Evaluation of different substrates for mass mltiplication of *Trichoderma* species. J. Pharmacogn. Phytochem..

[211] Andrzejak R., Janowska B. (2022). *Trichoderma* spp. improves flowering, quality, and nutritional status of ornamental plants. Int. J. Mol. Sci..

[212] Guzman-Guzman P., Kumar A., de los Santos-Villalobos S., Parra-Cota F.I., Orozco-Mosqueda M.D.C., Fadiji A.E., Hyder S., Babalola O.O., Santoyo G. (2023). *Trichoderma* species: Our best fungal allies in the biocontrol of plant diseases—A review. Plants.

[213] Zhou P., Cao J., Zhu H., Chen C., Lai Y., Zhang Y. (2023). Trichodermatides A-D, four new polyketides from *Trichoderma* sp. XM-3. Fitoterapia.

[214] Kassam R., Kranti K.V.V.S., Yadav J., Chatterjee M., Chawla G., Kundu A., Hada A., Thokala P.D., Shukla L., Mishra J. (2023). Exploration of rhizsophere-dwelling nematophagous *Trichoderma* spp. using novel bait technique with root-knot nematode *Meloidogyne incognita*. Biol. Control.

[215] Chan M.E., Tan J.Y., Lee Y.Y., Lee D., Fong Y.K., Mutwil M., Wong J.Y., Hong Y. (2023). Locally isolated *Trichoderma harzianum* species have broad spectrum biocontrol activities against the wood rot fungal species through both volatile inhibition and mycoparasitism. J. Fungi.

[216] De Oliveira C.M., Oshiquiri L.H., Almeida N.O., Steindorf A.S., Rocha M.R.D., Georg R.C., Ulhoa C.J. (2023). *Trichoderma harzianum* transcriptome in response to the nematode *Pratylenchus branchyurus*. Biol. Control.

[217] Wu J.-L., Yu Y.-H., Yao H.-Z., Zhao X., Yuan T., Huang Y.-H. (2023). Trichodermic acids from an endophytic *Trichoderma* sp. and their antifungal activity against the phytopathogen *Botrytis cinerea*. Phytochem. Lett..

[218] Tamandegani P.R., Sharifnabi B., Massah A., Zahravi M. (2021). Induced reprogramming of oxidative stress responses in cucumber by *Trichoderma asperellum* (Iran 3062C) enhances defense against cucumber mosaic virus. Biol. Control.

[219] Wang Z., Wang Z., Lu B., Quan X., Zhao G., Zhang Z., Liu W., Tian Y. (2022). Antagonistic potential of *Trichoderma* as a biocontrol agent *Sclerotinia asari*. Front. Microbiol..

[220] Bansal R., Sahoo S.A., Barvkar V.T., Srivastava A.K., Mukherjee P.K. (2023). *Trichoderma virens* exerts herbicidal effect on Arabidopsis thaliana via modulation of amino acid metabolism. Plant Sci..

[221] McLeod A., Labuschagne N., Kotze J. (1995). Evaluation of *Trichoderma* for biological control of avocado root rot in bark medium artificially infested with *Phytophthora cinnamomi*. S. Afr. Avocado Grow. Assoc. Yearb..

[222] Lopez-Lopez M.E., Del-Toro-Sanchez C.L., Gutierrez-Lomeli M., Ochoa-Ascencio S., Aguilar-Lopez J.A., Robles-Garcia M.A., Plascencia-Jatomea M., Bernal-Mercado M.A., Martinez-Cruz O., Avila-Novoa M.G. (2022). Isolation and characterization of *Trichoderma* spp. for antagonistic activity against Avocado (*Persea americana* Mill.) fruit pathogens. Horticulturae.

[223] Meki S., Ahmed S., Sakhuja P.K. (2011). Control of chickpea wilt (*Fusarium oxysporum* f.s.p. *ciceris*) using *Trichoderma* spp. in Ehtiopia. Arch. Phytopathol. Plant Prot..

[224] Rawat J., Sanwal P., Saxena J., Prasad R. (2023). Exploring the biochar as a suitable carrier for a bioinoculant *Aspergillus niger* K7 and its consequence on *Eleusine coracana* in field studies. J. Agric. Food Res..

[225] Olabiyi T.I., Ojo O.J., Adewuyi B.O., Moral M.T. (2016). Impact assessment of need compost and *Trichoderma harzianum* solution in the control of root knot nematode disease on cowpea. Cogent Food Agric..

[226] Gupta R., Singh M., Khan B.R. (2022). Photosynthetic electron transport rate and root dynamics of finger millet in response to *Trichoderma harzianum*. Plant Signal Behav..

[227] Choudhary A., Ashraf S., Musheer N. (2021). The antagonistic effect of locally isolated *Trichoderma* spp. against dry root rot of mungbean. Arch. Phytopathol. Plant Prot..

[228] Barbosa J.Z., Hungria M., Prior S.A., Moura M.C., Poggere G., Motta A.C.V. (2022). Improving yield and health of legume crops via co-inoculation with rhizobia and *Trichoderma*: A global meta-analysis. Appl. Soil Ecol..

[229] Pandey V., Ansari M.W., Tula S., Yadav S., Sahoo R.K., Shukla N., Bains G., Badal S., Chandra S., Gaur A.K. (2016). Does-dependent response of *Trichoderma harzianum* in improving drought tolerance in rice genotypes. Plants.

[230] Chowdhury M.R., Ahmed S.F., Khalid B., Bony Z.F., Asha J.F., Bhuiyan M.K.A. (2023). Biocontrol efficiency of microencapsulated *Trichoderma harzianum* coupled with organic additives against potato stem rot caused by *Sclerotium rolfsii*. Plant Stress.

[231] Limdolthamand S., Songkumarn P., Suwannarat S., Jantasorn A., Dethoup T. (2023). Bioconrol efficacy of endophytic *Trichoderma* spp. in fresh and dry powder formulations in controlling northern corn leaf blight in sweet corn. Biol. Control.

[232] Manzar N., Singh Y., Kashyap A.S., Sahu P.K., Rajawat M.V.S., Bhowmik A., Sharma P.K., Saxena A.K. (2021). Biocontrol potential of native *Trichoderma* spp. against anthracnose of great millet (*Sorghum bicolour* L.) from Tarai and hill regions of India. Biol. Control.

[233] Diaz-Gutierrez C., Arroyave C., Llugany M., Pschenrieder C., Martos S., Pelaez C. (2021). *Trichoderma asperellum* as a preventive and curative agent to control Fusarium wilt in *Stevia rebaudiana*. Biol. Control.

[234] Wang H., Tang W., Mao Y., Ma S., Chen X., Shen X., Yin C., Mao Z. (2020). Isolation of *Trichoderma virens* 6PS-2 and its effects on *Fusarium prolideratum* f. sp. *Malus domestica* MR5 related to apple replant disease (ARD) in China. Hortic. Plants J..

[235] Kishmoto K., Matsui K., Ozawa R., Takabayashi J. (2007). Volatile 1-Octen-3-Ol induced a defensive response in *Arabidopsis thaliana*. J. Gen. Plant Pathol..

[236] Contretas-Cornejo H.A., Macias-Rodriguez L., Herrera-Estrella A., Lopez-Bucio J. (2014). The 4-phosphopantetheinyl transferase of *Trichoderma virens* plays a role in plant protection against *Botrytis cinerea* through volatine organic compounds emission. Plant Soil.

[237] Lopez A.C., Alvarenga A.E., Zapata P.D., Luna M.F., Villalba L.L. (2019). *Trichoderma* spp. from misiones, Argentina: Effective fungi to promote plant growth of the regional crop *Ilex paraguariensis* St. Hil. Mycology.

[238] Ruano-Rosa D., Arjona-Girona I., Lopez-Herrera C.J.L. (2018). Integrated control of avocado white root rot combining low concentrations of fluazinam and *Trichoderma* spp.. Crop Prot..

[239] Muthukathan G., Mukherjee P., Salaskar D., Pachauri S., Tak H., Ganapathi T.R., Mukherjee P.K. (2020). Secretome of *Trichoderma virens* induced by banana roots-identification of novel fungal proteins for enhancing plant defence. Physiol. Mol. Plant Pathol..

[240] Almeida N.O., Oliveira C.M.D., Ulhoa C.J., Cortes M.V.D.C.B., Junior M.L., Rocha M.R.D. (2022). *Trichoderma harzianum* and *Trichoderma asperellum* are potential biocontrol agents of *Meloidogyne javanica* in banana cv. Grande Naine. Biol. Control.

[241] Sano L., Oliveira L.L.B.D., Leao M.D.M., Santos J.E.D.A.D., Medeiros S.C.D., Schneider F., Sousa A.B.O.D., Taniguchi C.A.K., Muniz C.R., Grangeiro T.B. (2022). *Trichoderma longibrachiatum* as a biostimulant of micropropagated banana seedlings under acclimatization. Plant Physiol. Biochem..

[242] Moreira F.M., Cairo P.A.R., Borges A.L., Silva L.D.D., Haddad F. (2021). Investigating the ideal mixture of soil and organic compound with *Bacillus* sp. and *Trichoderma asperellum* inoculations for optimal growth and nutrient content of banana seedlings. S. Afr. J. Bot..

[243] Costa A.C.D., Miranda R.F.D., Costa F.A., Ulhoa C.J. (2021). Potential of *Trichoderma piluliferum* as a biocontrol agent of *Colletotrichum musae* in banana fruits. Biocatal. Agric. Biotechnol..

[244] Mayo S., Gutierrez S., Malmierca M.G., Lorenzana A., Campelo M.P., Hermosa R., Casquero P.A. (2015). Influence of *Rhizoctonia solani* and *Trichoderma* spp. in growth of bean (*Phaseolus vulgaris* L.) and in the induction of plant defense-related genes. Front. Plant Sci..

[245] Eslahi N., Kowsari M., Motallebi M., Zamani M.R., Moghadasi Z. (2020). Influence of recombinant *Trichoderma* strains on growth of bean (*Phaseolus vulgaris* L.) by increased root colonization and induction of root growth related genes. Sci. Hortic..

[246] Silva F.D.A., Vieira V.D.O., Silva R.C.D., Pinheiro D.G., Soares M.A. (2021). Introduction of *Trichoderma* spp. biocontrol strains against *Sclerotinia scleotiorum* (Lib.) de Bary change soil microbial community composition in common bean (*Phaseolus vulgaris* L.) cultivation. Biol. Control.

[247] Ghoneem K.M., Al-Askar A.A., Saber W.-E.I.A. (2023). A simple formula of the endophytic *Trichoderma viride*, a case study for the management of *Rhizoctonia solani* on the common bean. Life.

[248] Abdelmoteleb A., Gonzalez-Mendoza D., Zayed O. (2023). Cell-free culture filtrate of Trichoderma longibrachiatum AD-1 as alternative approach to control *Fusarium solani* and induce defense response *Phaseolus vulgaris* L. plants. Rhizosphere.

[249] Bedine M.A.B., Iacomi B., Tchameni S.N., Sameza M.L., Fekam F.B. (2022). Harnessing the phosphate-solubilizing ability of Trichoderma strains to improve plant growth, phosphorus uptake and photosynthetic pigment contents in common bean (*Phaseolus vulgaris*). Biocatal. Agric. Biotechnol..

[250] Boat M.A.B., Sameza M.L., Iacomi B., Tchameni S.N., Boyom F.F. (2020). Screening, identification and evaluation of *Trichoderma* spp. for biocontrol potential of common bean damping-off pathogens. Biocontrol Sci. Technol..

[251] Silva F.D.A., Vierira V.D.O., Carrenho R., Rodrigues V.B., Junior M.L., Silva G.F.D., Soares M.A. (2021). Influence of the biocontrol agents *Trichoderma* spp. on the structure and functionality of the edaphic microbial community in common bean cultivars (*Phaseolus vulgaris* L.) inoculated with *Sclerotinia sclerotiorum* (Lib.) de Bary. Appl. Soil Ecol..

[252] Umadevi P., Anandaraj M., Srivastav V., Benjamin S. (2018). *Trichoderma harzianum* MTCC 5179 impacts the population and functional dynamics of microbial community in the rhzisophere of black pepper (*Piper nigrum* L.). Brazil J. Microbiol..

[253] Pradhan D.A., Makandar R. (2023). Delineating host responses induced by *Trichoderma* in castor through comparative transcriptome analysis. Rhizosphere.

[254] Prasad R.D., Chandrika K.S.V.P., Godbole V. (2020). A novel chitosan biopolymer based *Trichoderma* delivery system: Storage stability, persistence and bio efficacy against seed and soil borne diseases of oilseed crops. Microbiol. Res..

[255] Verma R., Das A., Kaman P.K., Kushwaha K.P.S., Bisht A.S., Narzary P.R., Roy B. (2023). Comparative evaluation of physiological and biochemical chages in black pepper plants infected by *Colletotrichum siamense* in response to *Trichoderma viride* and *T. harzianum* application. Physiol. Mol. Plant Pathol..

[256] Tchameni S.N., Ngonkeu M.E.L., Begoude B.A.D., Nana L.W., Fokom R., Owona A.D., Mbarga J.B., Tchana T., Tondje P.R., Etoa F.X. (2011). Effect of *Trichoderma asperellum* and arbuscular mycorrhizal fungi on cacao growth and resistance against black pod disease. Crop Prot..

[257] Mbarga J.B., Begoude B.A.D., Ambang Z., Meboma M., Kuate J., Ewbank W., Hoopen G.M.T. (2020). Field testing an oil-based *Trichoderma asperellum* formulation for the biological control of cacao black pod disease caused by *Phytophthora megakarya*. Crop Prot..

[258] Mbargo J.B., Begoude B.A.D., Ambang Z., Meboma M., Kuate J., Schiffers B., Ewbank W., Dedieu L., Hoopen G.M.T. (2014). A new oil-based formulation of *Trichoderma asperellum* for the biological control of cacao black pod disease caused by *Phytophthora megakarya*. Biol. Control.

[259] Motlagh M.R.S., Abolghasemi M. (2022). The effect of *Trichoderma* spp. isolates on some morphological traits of canola inoculated with *Sclerotinia sclerotiorum* and evaluation of their efficacy in biological control of pathogen. J. Saudi Soc. Agric. Sci..

[260] Da Silva J.S.A.D., Medeiros E.V.D., Costa D.P.D., Souza C.A.F.D., Oliveira J.B.D., Franca R.F.D., Souza-Motta C.M., Lima J.R.D.S., Hammecker C. (2022). Biochar and *Trichoderma aureoviride* URM 5158 as alternatives for the management of cassava root rot. Appl. Soil Ecol..

[261] Pradham D.A., Bagagoni P., Slathia S., Prasad R.D. (2023). Characterization of *Trichoderma* strains for novel species-specific markers by multiplex PCR and antagonistic property against *Alternaria ricini* in castor (*Ricinus communis* L.). Biocatal. Agric. Biotechnol..

[262] Pradhan D.A., Bagagoni P., Makandar R. (2023). Assessing rhizosphere *Trichoderma asperellum* strains for root colonizing and antagonistic competencies against *Fusarium* wilt through molecular and biochemical responses in castor. Biol. Control.

[263] Dubey S.C., Tripathi A., Bhavani R., Singh B. (2011). Evaluation of seed dressing and soil application formulation of *Trichoderma* species for integrated management of dry root rot of chickpea. Biocontrol. Sci. Technol..

[264] Begum M.F., Rahman M.A., Firoz Alam M. (2010). Biological control of *Alternaria* fruit rot of chili by *Trichoderma* species under field conditions. Mycobiology.

[265] Ji S., Liu Z., Liu B., Wang Y., Wang J. (2020). The effect of *Trichoderma* biofertilizer on the quality of flowering Chinese cabbage and the soil environment. Sci. Hortic..

[266] De Lima F.B., Felix C., Osorio N., Alves A., Vitorino R., Domingues P., da Silva Ribeiro R.T., Esteves A.C. (2017). *Trichoderma harzianum* T1A constitutively secretes proteins involved in the biological control of *Guignardia citricarpa*. Biol. Control.

[267] Bae H., Sicher R.C., Kim M.S., Kim S.H., Strem M.D., Melnick R.L., Bailey B.A. (2009). The beneficial endophyte *Trichoderma hamatum* isolate DIS 219b promotes growth and delays the onset of the drought response in *Theobroma cacao*. J. Exp. Bot..

[268] Wang R., An X., Lv Y., Khan R.A.A., Xue M., Chen J., Liu T. (2023). *Trichoderma asperellum* GD040 upregulates defense-related genes and reduces lesion size in *Coffea canephora* leaves inoculated with *Colletotrichum cairnsense*. Biol. Control.

[269] Howell C.R., Hanson L.E., Stipanovic R.D., Puckhaber L.S. (2000). Induction of terpenoid synthesis in cotton roots and control of *Rhizoctonia solani* by seed treatment with *Trichoderma virens*. Phytopathology.

[270] Howell C.R. (2002). Cotton seedling preemergence damping-off incited by *Rhizopus oryzae* and *Pythium* spp. and its biological control with *Trichoderma* spp.. Phytopathology.

[271] Howell C.R. (2006). Understanding the mechanisms employed by *Trichoderma virens* to effect biological control of cotton diseases. Phytopathology.

[272] Larran S., Simon M.R., Santamarina M.P., Caselles J.R., Consolo V.F., Perello A. (2023). Endophytic *Trichoderma* strains increase soya bean growth and promote charcoal rot control. J. Saudi Soc. Agric. Sci..

[273] Nakkeeran S., Renukadevi P., Marimuthu T. (2005). Antagonistic potentiality of *Trichoderma viride* and assessment of its efficacy for the management of cotton root rot. Arch. Phytopathil. Plant Prot..

[274] Gajera H.P., Hirpara D.G., Savaliya D.D., Golakiya B.A. (2020). Extracellular metabolomics of *Trichoderma* biocontroller for antifungal action to restrain *Rhizoctonia solani* Kuhn in cotton. Physiol. Mol. Plant Pathol..

[275] Oliveira C.M.D., Almeida N.O., Cortes M.V.D.C.B., Junior M.L., Rocha M.R.D., Ulhoa C.J. (2021). Biological control of *Pratylenchus brachyurus* with isolates of *Trichoderma* spp. on soybean. Biol. Control.

[276] Szczech M., Nawrocka J., Felczynski K., Malolepsza U., Sobolewski J., Kowalska B., Maciorowski R., Jas K., Kancelista A. (2017). *Trichoderma atroviride* TRS25 isolate reduces downy mildew and induces systemic defence responses in cucumber in field conditions. Sci. Hortic..

[277] Nawrocka J., Malolepsza U., Szymczak K., Szczech M. (2018). Involvement of metabolic components, volatile compounds, PR proteins and mechanical strengthening in multilayer protection of cucumber plants against *Rhizoctonia solani* activated by *Trichoderma atroviride* TRS25. Protoplasma.

[278] Yuan M., Huang Y., Ge W., Jia Z., Song S., Zhang L., Huang Y. (2019). Involvement of jasmonic acid, ethylene and salicylic acid signaling pathways behind the systemic resistance induced by *Trichoderma longibrachiatum* H9 in cucumber. BMC Genom..

[279] Zhao L., Zhang Y. (2015). Effects of phosphate solubilization and phytohormone production of *Trichoderma asperellum* Q1 on promoting cucumber growth under salt stress. J. Integr. Agr..

[280] Brotman Y., Landau U., Cuadros-Inostroza A., Takayuki T., Fernie A.R., Chet I., Viterbo A., Willmitzer L. (2013). *Trichoderma*-plant root colonization: Escaping early plant defense responses and activation of the antioxidant machinery for saline stress tolerance. PLOS Pathog..

[281] Wang R., Yu X., Yi Y., Norvienyeku J., Khan R.A.A., Zhang M., Ren S., Chen J., Liu T. (2023). Biocontrol of cucumber Fuarium wilt by *Trichoderma asperellum* FJ035 dependent on antagonism and spatiotemporal competition with *Fusarium oxysporum*. Biol. Control.

[282] Li P., Xie D., Chen H., Qiu Y., Zhang X., Zhang S., Wang L., Lin H., Li X., Liu K. (2023). Secondary metabolites from marine derived fungus *Penicillium chrysogenum* Y19-1 with proangiogenic and antithrombotic activities. Biochem. System. Ecol..

[283] Ulrich A., Lerin L.A., Camargo A.F., Scapini T., Diering N.L., Bonafin F., Gasparetto I.G., Confortin T.C., Sansonovicz P.F., Fabaian R.L. (2021). Alternative bioherbicide based on *Trichoderma koningiopsis*: Enzymatic characterization and its effect on cucumber plants and soil organism. Biocatal. Agric. Biotechnol..

[284] Intana W., Kumla J., Suwannarach N., Sunpapao A. (2023). Biological control potential of a soil fungus *Trichoderma asperellum* K1-02 against *Neoscytalidium dimidiatum* causing stem canker of dragon fruit. Physiol. Mol. Plant Pathol..

[285] Admasu W., Sintayehu A., Gezahgne A., Terefework Z. (2023). In vitro bioefficacy of *Trichoderma* species against two *Botryosphaeriaceae* fungi causing *Eucalytpus* stem canker disease in Ethiopia. J. Nat. Pest Res..

[286] Mbazia A., Youssef N.O.B., Kharrat M. (2016). Tunisian isolates of *Trichoderma* spp. and *Bacillus subtilis* can control *Botrytis fabae* on faba bean. Biocontrol Sci. Technol..

[287] Neme A., Leta A., Yones A.M., Tahir M. (2023). Seedborne mycoflora of faba bean (*Vicia fabae* L.) and evaluation of plant extract and *Trichoderma* species against mycelium growth of selected fungi. Heliyon.

[288] Gupta V.P., Sharma D.D., Mahasevaswamy H., Chandrashekar D.S. (2009). *Trichoderma pseudokoningii* for hastening the decomposition of various sericultural wastes and impact of enriched composts on disease suppression in mulberry (*Morus* spp.). Arch. Phytopathol. Plant Prot..

[289] Brito F.D.S., Costa D.P.D., Souza C.A.F.D., Almeida D.T.D.R.G.F.D., Leite I.C.H.D.L., Goncalves E.P., Medeiros E.V.D. (2022). Selection and control efficacy of *Trichoderma* spp. against *Fusarium solani* and *Lasiodiplodia theobromae* causing root rot in forage cactus. Physiol. Mol. Plant Pathol..

[290] Mastan A., Rane D., Dastager S.G., Vivek Babu C.S. (2021). Molecular insights of fungal endophyte co-inoculation with *Trichoderma viride* for the augmentation of forskolin biosynthesis in *Coleus forskohlii*. Phytochemistry.

[291] Kupper V., Kortekamp A., Steiner U. (2023). Combining *Trichoderma koningiopsis* and chitosan as a synergistic biocontrol and biostimulating complex to reduce copper rates for downy mildew control on grapevine. Biol. Control.

[292] El-Mohamedy R., Ziedan E.H., Abdalla A.M. (2010). Biological soil treatment with *Trichoderma harzianum* to control root rot disease grapevine (*Vitis vinifera* L.) in newly reclaimed lands in Bobaria province. Arch. Phytopathol. Plant Prot..

[293] Csoto A., Kovacs C., Pal K., Nagy A., Peles F., Fekete E., Karaffa L., Kubicek C.P., Sandor E. (2023). The biocontrol potential of endophytic *Trichoderma* fungi isolated from Hungarian grapevines, part II, grapevine stimulation. Pathogens.

[294] Song H., Asghari M., Zahedipour-Sheshglani P., Alizadeh M., Qian S., Diao E. (2023). Modeling and optimizing the effects of *Trichoderma* on quality, decay extension rate and phytochemical compounds of Thompson seedless table grapes by the use of response surface methodology. Eur. J. Agron..

[295] Gajera H.P., Savaliya D.D., Patel S.V., Golakiya B.A. (2015). *Trichoderma viride* induces pathogenesis related defense response against rot pathogen infection in groundnut (*Arachis hypogaea* L.). Infect Gene Evol..

[296] Gajera H.P., Katakpara Z.A., Golakiya B.A. (2016). Antioxidant defense response induced by *Trichoderma viride* against *Aspergillus niger* Van Tieghem causing collar rot in groundnut (*Arachis hypogaea* L.). Microbiol. Pathogen..

[297] Ayyandurai M., Akila R., Manonmani K., Harish S., Mini M.L., Vellaikumar S. (2023). Deciphering the mechanism of *Trichoderma* spp. consortia possessing volatile organic compounds and antifungal metabolites in the suppression of *Sclerotium rolfsii* in groundnut. Physiol. Mol. Plant Pathol..

[298] Hoa P.T.B., Tue N.H., Trang H.T.Q., Thu H.A., Nhung L.N.H., Luong N.N., Huy N.X., Tien N.Q.D., Loc N.H. (2023). Enhancement of resistance against fungal pathogens in peanut (*Arachis hypogaea* L.) cultivar L14 by heterologous expression of gene encoding chitinase 42 kDa from *Trichoderma asperellum* SH16. S. Afr. J. Bot..

[299] Boukaew S., Petlamul W., Srinuanpan S., Nooprom K., Zhang Z. (2024). Heat stability of *Trichoderma asperelloides* SKRU-01 culture filtrates: Potential applications for controlling fungal spoilage and AFB_1_ production in peanuts. Int. J. Food Microbiol..

[300] Erazo J.G., Palacios S.A., Pastor N., Giordano F.D., Rovera M., Reynoso M.M., Venisse J.S., Torres A.M. (2021). Biocontrol mechanisms of *Trichoderma harzianum* ITEM 3636 against peanut brown root rot caused by *Fusarium solani* RC 386. Biol. Control.

[301] Yan D., Cai N., Nong X., Wang G., Wang Q., Ullah H., Tu X., Zhang Z. (2022). Transcriptomic differences in response to *Metarhizium anisopliae* and *Trichoderma harzianum* uncovers major regulative genes and pathways for establishment of beneficial relationship in peanut. Biol. Control.

[302] Song X., Jin J., Yin H., Wang T., Zong H., Wang F., Liu J., Huang X., Wang B., Chai C. (2023). *Trichoderma viride* F7 improves peanut performance while remedying cadmium-contaminated soil with microplastics. Pedosphere.

[303] Sennoi R., Singkham N., Jogloy S., Boonlue S., Saksirirat W., Kesmala T., Patanothai A. (2013). Biological control of southern stem rot caused by *Sclerotium rolfsii* using *Trichoderma harzianum* and arbuscular mycorrhizal fungi on Jerusalem artichoke (*Helianthus tuberosus* L.). Crop Prot..

[304] Silva L.R.D., Valadares-Inglis M.C., Peixoto G.H.S., Luccas B.E.G.D., Muniz P.H.P.C., Magalhaes D.M., Moraes M.C.B., Mello S.C.M.D. (2021). Volatile organic compounds emitted by *Trichoderma azevedoi* promote the growth of lettuce plants and delay the symptoms of white mold. Biol. Control.

[305] Wonglom P., Ito S.-I., Supapao A. (2020). Volatile organic compounds emitted from endophytic fungus *Trichoderma asperellum* T1 mediate antifungal activity, defense response and promote plant growth in lettuce (*Lactuca sativa*). Fungal Ecol..

[306] Baiyee B., Pornsuriya C., Ito S.-I., Sunpapao A. (2019). *Trichoderma spirale* T76-1 displays biocontrol activity against leaf spot on lettuce (*Lactuca sativa* L.) caused by *Corynespora cassiicola* or *Curvularia aeria*. Biol. Control.

[307] Caporale A.G., Sommella A., Lorito M., Lombardi N., Azam S.M.G.G., Pigna M., Ruocco M. (2014). *Trichoderma* spp. alleviate phytotoxicity in lettuce plants (*Lactuca sativa* L.) irrigated with arsenic-contaminated water. J. Plant Physiol..

[308] Santos-Villalobos S., Guzman-Ortiz D.A., Gomez-Lim M.A., Delano-Frier J.P., De-Folter S., Sanchez-Garcia P., Pena-Cabriales J.J. (2013). Potential use of *Trichoderma asperellum* (Samuels, Liechfeldt et Nirenberg) T8a as a biological control agent against anthracnose in mango (*Mangifera indica* L.). Biol. Control.

[309] Zhan X., Khan R.A.A., Zhang J., Chen J., Yin Y., Tang Z., Wang R., Lu B., Liu T. (2023). Control of postharvest stem-end rot on mango by antifungal metabolites of *Trichoderma pinnatum* LS2029-3. Sci. Hortic..

[310] Akladious S.A., Abbas S.M. (2014). Application of *Trichoderma harzianum* T22 as a biofertilizer potential in maize growth. J. Plant Nutr..

[311] Yassin M.T., Mostafa A.A.-F., Al-Askar A.A. (2022). In vitro antagonistic activity of *Trichoderma* spp. against fungal pathogens causing black point disease of wheat. J. Taibah Univ. Sci..

[312] Degani O., Dor S. (2021). *Trichoderma* biological control to protect sensitive maize hybrids against Late wilt disease in the field. J. Fungi.

[313] Yu C., Luo X. (2020). *Trichoderma koningiopsis* controls *Fusarium oxysporum* causing damping-off in *Pinus massoniana* seedlings by regulating active oxygen metabolism osmotic potential, and the rhizosphere microbiome. Biol. Control.

[314] Saravanakumar K., Wand M.H. (2020). Isolation and molecular identification of *Trichoderma* species from wetland soil and their antagonistic activity against phytopathogens. Physiol. Mol. Plant Pathol..

[315] Karuppiah V., Li Y., Sun J., Vallikkannu M., Chen J. (2020). Vel1 regulates the growth of *Trichoderma atroviride* during co-cultivation with *Bacillus amyloliquefaciens* and is essential for wheat root rot control. Biol. Control.

[316] Galletti S., Paris R., Cianchetta S. (2020). Selected isolates of *Trichoderma gamsii* induce different pathways of systemic resistance in maize upon *Fuarium verticillioides* challenge. Micriobiol. Res..

[317] Hasanloo T., Kowsari M., Naraghi S.M., Bagheri O. (2010). Study of different *Trichoderma* strains on growth characteristics and silymarin accumulation of milk thistle plant. J. Plant Interact..

[318] Hohmann P., Jones E.E., Hill R.A., Stewart A. (2011). Understanding *Trichoderma* in the root system of *Pinus radiata*: Associations between rhizosphere colonisation and growth promotion for commercially growth seedlings. Fungal Biol..

[319] Hohmann P., Jones E.E., Hill R.A., Stewart A. (2012). Ecological studies of the bio-inoculant *Trichoderma hamatum* LU592 in the root system of *Pinus radiata*. FEMS Microbiol. Ecol..

[320] Ruangwong Q.-U., Wonglom P., Phoka N., Suwannarach N., Lumyong S., Ito S.-I., Sunpapao A. (2021). Biological control activity of *Trichoderma asperelloides* PSU-P1 against gummy stem blight in muskmelon (*Cucumis melo*). Physiol. Mol. Plant Pathol..

[321] Khare A., Singh B., Upadhyay R. (2010). Biological control of *Pythium aphanidermatum* causing damping-off of mustard by mutants of *Trichoderma viride* 1433. J. Agric. Technol..

[322] Samlikamnoed P., Anothai J., Chairin T. (2023). Defense-related enzyme production in oil palm seedlings against basal stem rot pathogen *Ganoderma boninense* and its biological control by *Trichoderma asperellun*. Physiol. Mol. Plant Pathol..

[323] Ben Amira M., Lopez D., Triki Mohamed A., Khouaja A., Chaar H., Fumanal B., Gousset-Dupont A., Bonhomme L., Label P., Goupil P. (2017). Beneficial effect of *Trichoderma harzianum* strain Ths97 in biocontrolling *Fusarium solani* causal agent of root rot disease in olive trees. Biol. Control.

[324] Metwally R.A., Soliman S.A., Laref A.A.H.A., Abdelhameed R.E. (2021). The individual and interactive role of arbuscular mycorrhizal fungi and *Trichoderma viride* on growth, protein content, amino acids fraction, and phosphatases enzyme activities of onion plant amended with fish waste. Ecotoxicol. Environ. Saf..

[325] Vargas J.T., Rodriguez-Monroy M., Meyer M.L., Montes-Belmont R., Sepulveda-Jimenez G. (2017). *Trichoderma asperellum* ameliorates phytotoxic effects of copper in onion (*Allium cepa* L.). Environ. Exp. Bot..

[326] Camacho-Luna V., Pizar-Quiroz A.M., Rodriguez-Hernandez A.A., Rodriguez-Monroy M., Sepulveda-Jimenez G. (2023). *Trichodera longibrachiatum*, a biological control agent of *Sclerotium cepivorum* on onion plants under salt stress. Biol. Control.

[327] Da Silva L.R., Rodrigues L.L.D.B., Zazaroni A.B., Castro B.S.D., Sifuentes D.N., Botelho A.S., Moraes M.C.B., Mello S.C.M.D. (2022). *Sclerotium rolfssi* mycelial profile analysis by MALDI-TOF related to biological control, volatile organic compounds diversity and onion growth promotion, as influenced by *Trichoderma* spp.. Biol. Control.

[328] Bunbury-Blanchette A.L., Walker A.K. (2019). *Trichoderma* species show biocontrol potential in dual culture and greenhouse bioassays against Fusarium basal rot of onion. Biol. Control.

[329] Arif S., Munis M.F.H., Liaquat F., Gulzar S., Haroon U., Zhao L., Zhang Y. (2023). *Trichoderma viride* establishes biodefense against clubroot (*Plasmodiophora brassicae*) and fosters plant growth via colonizing root hairs in pak choi (*Brassica campestris* spp. *chinensis*). Biol. Control.

[330] Gulzar S., Manzoor M.A., Liaquat F., Shah I.H., Rehman A., Hameed M.K., Arif S., Zhou X., Zhang Y. (2023). Effects of melatonin and *Trichoderma harzianum* on pak choi yield, chlorophyll contents and antioxidant defense system under clubroot disease. S. Afr. J. Bot..

[331] Nandini B., Hariprasad P., Niranjana S.R., Shetty H.S., Geetha N.P. (2013). Elicitation of resistance in pearl millet by oligosaccharides of *Trichoderma* spp. against downy mildew disease. J. Plant Inter..

[332] Nandini B., Geetha N., Prakash H.S., Hariparsad P. (2021). Natural uptake of anti-oomycetes *Trichoderma* produced secondary metabolites from pearl millet seedlings- A new mechanism of biological control of downy mildew disease. Biol. Control.

[333] Rochal K.K.L., Pierre E., Diane Y.Y., Sahu K.P., Vanessa N.D., Wankeu K., Herman T., Gilbert G.T.P., Sevilor K., Germain K. (2021). Biological elicitor potential of endospheric *Trichoderma* and derived consortia against *pepper* (*Capsicum annuum* L.) *leaf curl virus*. Arch. Phytopathol. Plant Prot..

[334] Eke P., Dinango V.N., Wakam L.N., Toghueo R.M.K., Kouokap L.R.K., Mabou L.C.N., Wankeu T.H.K., Ngomsi P., Boyom F.F. (2020). Diagnosis and bioefficiancy of endospheric *trichoderma* strains of selected medicinal plant on pepper root rot and vascular wilt in Cameroon. Arch. Phytopathol. Plant Prot..

[335] Demissie S., Megersa G., Meressa B.H., Muleta D. (2021). Resistance levels of Ethiopian hot pepper (*Capsicum* spp.) varieties to a pathogenic *Fusarium* spp. and in vitro antagonistic effect of *Trichoderma* spp.. Arch. Phytopathol. Plant Prot..

[336] Pereira T.D.S., Macedo A.G., Silva J.D., Pinheiro J.B., Paula A.M.D., Biscaia D., Busato J.G. (2020). Water-extractable fraction of vrmicomposts enriched with *Trichoderma* enhances the growth of bell pepper and tomato as well as their tolerance against *Meloidogyne incognita*. Sci. Hortic..

[337] Tomah A.A., Alamer I.S.A., Li B., Zhang J.-Z. (2020). A new species of *Trichoderma* and gliotoxin role: A new observation in enhancing biocontrol potential of *T. virens* against *Phytophthora capsici* on chili pepper. Biol. Control.

[338] Rokni N., Alizadeh H.S., Bazgir E., Darvishnia M., Mirzaei-Najafgholi H. (2021). The tripartite consortium of *Serendipita indica*, *Trichoderma simmonsii*, and bell pepper (*Capsicum annum*). Biol. Control.

[339] Guo K., Sui Y., Li Z., Huang Y., Zhang H., Wang W. (2020). Colonization of *Trichoderma viride* Tv-1511 in perppermint (*Mentha* × *piperita* L.) roots promotes essential oil production by triggering ROS-mediated MAPK activation. Plant Physiol. Biochem..

[340] Mishra R.K., Pandey S., Hazra K.K., Mishra M., Naik S.J.S., Bohra A., Parihar A.K., Rathore U.S., Naimuddin K.K., Singh B. (2023). Biocontrol efficacy and induced defense mechanisms of indigenous *Trichoderma* strains against *Fusarium* wilt (*F. udum* (Butler) in pigeonpea. Physiol. Mol. Plant Pathol..

[341] Dehriya K., Shukla A., Ganaie M.A., Vyas D. (2015). Individual and interactive role of *Trichoderma* and Mycorrhizae in controlling wilt disease and growth reduction in *Cajanus cajan* caused by *Fusarium udum*. Arch. Phytopathol. Plant Prot..

[342] Kelley W.D. (1977). Interactions of *Phytophthora cinnamomi* and *Trichoderma* spp. in relation to propagule production in soil cultures at 26 °C. Can. J. Microbiol..

[343] Mollah M.M.I., Hassan N. (2023). Efficacy of *Trichoderma harzianum*, as a biological fungicide against fungal diseases of potato, late blight and early blight. J. Nat. Pest Res..

[344] Hassan M.M., Soliman M.M., Al-Otaibi S., El-Shehawi A.M., Taha E.-K.A., Sayed S. (2022). The effectiveness of *Xanthium strumamrium* L. extract and *Trichoderma* spp. against pomegranate isolated. J. King Saud. Univ. Sci..

[345] Metz N., Hausladen H. (2022). *Trichoderma* spp. as potential biological control agent against *Alternaria solani* in potato. Biol. Control.

[346] Ommati F., Zaker M. (2012). In vitro and greenhouse evaluations of *Trichoderma* isolates for biological control of potato wilt disease (*Fusarium solani*). Arch. Phytopathol. Plant Prot..

[347] Sivakumar D., Wijeratnam R.S., Wijesundera R.L., Marikar F.M., Abeyesekere M. (2000). Antagonistic effect of *Trichoderna harzianm* on postharvest pathogens of rambutan (*Nephelium lappaceum*). Phytoparasitica.

[348] Pandey A.K., Kumar A., Samota M.K., Tanti A. (2022). *Trichoderma reesei* as an elicitor triggers defense responses in tea plant and delays gray blight symptoms. Pest Biochem. Physiol..

[349] Klaram R., Jantasorn A., Dethoup T. (2022). Efficacy of marine antagonist, *Trichoderma* spp. as halo-tolerant biofungicide in controlling rice diseases and yield improvement. Biol. Control.

[350] Chen L.-H., Zhang J., Shao X.-H., Wang S.-S., Miao Q.-S., Mao X.-Y., Zhai Y.-M., She D.-L. (2015). Development and evaluation of *Trichoderma asperellum* preparation for control of sheath blight of rice (*Oryza sativa* L.). Biocontrol Sci. Technol..

[351] Parizi T.E., Ansari M., Elaminejad T. (2012). Evaluation of the potential of *Trichoderma viride* in the control of fungal pathogens of Roselle (*Hibiscus sabdariffa* L.) in vitro. Microbiol. Pathogen..

[352] Elad Y., Chet I., Boyle P., Henis Y. (1983). Parasitism of *Trichoderma* spp. on *rhizoctonia solani* and *Sclerotium rolfsii*-scanning electron microscopy and fluorescence microscopy. Phytopathology.

[353] Vecstaudza D., Grantina-levina L., Makarenkova G., Kasparinskis R., Selga T., Steinberga V., Stelmahere S., Steiner C., Muter O. (2018). The impact of wood-derived biochar on the survival of *Trichoderma* spp. and growth of *Secale cereale* L. in sandy soil. Biocontrol Sci. Technol..

[354] Arellano A.D.V., Dilva G.M.D., Guatimosim E., Dorneles K.D.R., Moreira L.G., Dallagnol L.J. (2021). Seeds coated with *Trichoderma atroviride* and soil amended with silicon improve the resistance of *Lolium multiflorum* against *Pyricularia oryzae*. Biol. Control.

[355] John R.P., Tyagi R.D., Prevost D., Brar S.K., Pouleur S., Surampalli R.Y. (2010). Mycoparasitic *Trichoderma viride* as a biocontrol agent against *Fusarium oxysporum* f. sp. adzuki and *Pythium arrhenomanes* and as a growth promoter of soybean. Crop Prot..

[356] Conte E.D., Magro T.D., Bem L.C.D., Dalmina J.C., Matte J.A., Schenkel V.O., Schwambach J. (2022). Use of *Trichoderma* spp. in no-tillage system: Effect of soil and soybean crop. Biol. Control.

[357] Ulrich A., Muller C., Gasparetto I.G., Bonafin F., Diering N.L., Camargo A.F., Reichert Junior F.W., Paudel S.R., Treichel H., Mossi A.J. (2023). Bioherbicide effects of *Trichoderma koningiopsis* associated with commercial formulations of glyphosate in weeds and soybean plants. Crop Prot..

[358] Camatti G., Santos F.M.D., Rodrigues Junior G.L.D.S., Camargo D.P., Mandio G.S., Santos J.R.P., Silva J.C.P.D. (2023). *Bacillus*- and *Trichoderma*-based products control the spiral nematode *Helicotylenchus dihystera* in soybean. Rhizosphere.

[359] Gashash E.A., El-Sayed S.A., Kamel W.M., Sarhan E.A.D. (2023). Evaluation of the potential of native *Trichoderma* spp. against root-rot diseases of soybean. Arch. Phytopathol. Plant Prot..

[360] Sofy M., Mohamed H., Dawood M., Abu-Elsaoud A., Soliman M. (2022). Integrated usage of *Trichoderma harzianum* and biochar to ameliorate salt stress on spinach plants. Arch. Agron. Soil Sci..

[361] Naher L., Yusuf U.K., Ismail A., Hossain K. (2014). *Trichoderma* spp.: A biocontrol agent for sustainable managment of plant diseases. Pak. J. Bot..

[362] Qiu Y.-L., Wang N., Zhang J.-F., Chen Z.-H., Yan Y.-T., Wu F., Li H.-L. (2021). Application of indigenous honeybees in dispersing *Trichoderma harzianum* spores for control of the strawberry grey mould. Biocontrol Sci. Technol..

[363] Kappel L., Kosa N., Gruber S. (2022). The multilateral efficacy of chitosan and *Trichoderma* on sugar beet. J. Fungi.

[364] Paramasivan M., Chandrasekaran A., Mohan S., Muthukrishnan N. (2014). Ecological management of tropical sugar beet (TSB) root rot (*Sclerotium rolfsii* (Sacc.) by rhizosphere *Trichoderma* species. Arch. Phytopathol. Plant Prot..

[365] Upadhyay J.P., Mukhopadhyay A.N. (1986). Biological control of *Sclerotium rolfsii* by *Trichoderma harzianum* in sugarbeet. Trop Pest Manag..

[366] Kakvan N., Heydari A., Zamanizadeh H.R., Rezaee S., Naraghi L. (2013). Development of new bioformulations using *Trichoderma* and *Talaromyces* fungal antagonists for biological control of sugar beet damping-off disease. Crop Prot..

[367] Shukla S.K., Jaiswal V.P., Sharma L., Tiwari R., Pathak A.D., Gaur A., Awasthi S.K., Srivastava A. (2022). Trash management and *Trichoderma harzianum* influencing photosynthesis, soil carbon sequestration, and growth and yield of sugarcane ratoon in subtropical India. Eur. J. Agron..

[368] Devi S.S., Sreenivasulu Y., Saritha S., Kumar M.R., Kumar K.P., Sudharak P. (2012). Molecular diversity of native *Trichoderma* isolates against *Fusarium oxysporum* f. sp. *lycopersici* (Sacc.). A causal agent of *Fusarium* wilt in tomato (*Lycopersicon esculentum* Mill.). Arch. Phytopathol. Plant Prot..

[369] Mushtaq S., Bareen F., Tayyeb A., Nazir A. (2023). Autochthonous strains of *trichoderma* isolated from tannery solid waste improve phytoextraction potential of heavy metals by sunflower. Int. J. Phytoremed..

[370] Arzanlou M., Khodaei S., Narmani A., Babai-Ahari A., Azar A.M. (2014). Inhibitory effect of *Trichoderma* isolates on growth of *Alternaria alternata*, the causal agent of leaf spot disease on sunflower, under laboratory conditions. Arch. Phytopathol. Plant Prot..

[371] Yadav A., Yadva K., Aggarwal A. (2015). Impact of arbuscular mycorrhizal fungi with *Trichoderma viride* and *Pseudomonas flurescens* on growth, yield, and oil content in *Helianthus annuus* L.. J. Essent Oil Bear Plants.

[372] Mathews J.R., Sivparsad B.J., Laing M.D. (2019). Greenhouse evaluation of *Trichoderma harzianum* for the control of *Sclerotinia* wilt (*Sclerotinia sclerotiorum*) of sunflower. S. Afr. J. Plant Soil.

[373] Ozer N., Coskuntuna A., Sabudak T. (2021). *Trichoderma harzianum*-induced defense in sunflower (*Helianthus annuus* L.) against *Plasmopara halstedii* with changes in metabolite profiling of roots. Biocontrol Sci. Technol..

[374] Wang X., Xu S., Wu S., Feng S., Bai Z., Zhuang G., Zhuang X. (2018). Effect of *Trichoderma viride* biofertilizer on ammonia volatilization from an alkaline soil in Northern China. J. Environ. Sci..

[375] Li X., Han S., Wang G., Liu X., Amombo E., Xie Y., Fu J. (2017). The fungus *Aspergillus aculeatus* enhances salt-stress tolerance, metabolite accumulation, and improves forage quality in perennial ryegrass. Front. Microbiol..

[376] Andrzejak R., Janowska B. (2022). Flowering, nutritioal status, and content of chloroplast pigments in leaves of *Gladiolus hybridus* L. Advances Red after application of *Trichoderma* spp.. Sustainability.

[377] Pandey A.K., Samota M.K., Tanti A.J., Babu A. (2023). *Trichoderma reesei* induces defense-related biochemical markers associated with resistance to Fusarim dieback in tea crop. Biol. Control.

[378] Zhang S., Zhang C., Gao Z.-F., Qiu C.-W., Shi S.-H., Chen Z.-H., Ali M.A., Wang F., Wu F. (2023). Integrated physiological and omics analyses reveal the mechanism of beneficial fungi *Trichoderma* sp. alleviating cadmium toxicity in tobacco (*Nicotiana tabacum* L.). Ecotoxicol. Environ. Saf..

[379] Khalil M.I.I., Youssef S.A., Tartoura K.A., Eldesoky A.A. (2021). Comparative evaluation of physiological and biochemical alteration in tomato plants infected by *Alternaria alternata* in response to *Trichoderma viride* and *Chaetomium globosum* application. Physiol. Mol. Plant Pathol..

[380] Olowe O.M., Nicola L., Asemoloye M.D., Akanmu A.O., Babalola O.O. (2022). *Trichoderma*: Potential bio-resource for the management of tomato root rot diseases in Africa. Microbiol. Res..

[381] Devi S.S., Sreenivasulu Y., Rao K.V.B. (2017). Protective role of *Trichoderma logibrachiatum* (WT2) on lead induced oxidative stress in *Helianthus annus* L.. Indian J. Exp. Biol..

[382] Singh J., Kumar V., Srivastava S., Kumar A., Singh V.P. (2018). In vitro evaluation of *Trichoderma* species against *Fusarium oxysporum* f. sp. lycopersici causing tomato wilt. Plant Pathol. J..

[383] Zehra A., Aamir M., Dubey M.L., Ansari W.A., Meena M., Swapnil P., Upadhyay R.S., Ali M.A., Al-Ghamdi A.A., Lee J. (2023). Enhanced protection of tomato against *Fusarium* wilt through biopriming with *Trichoderma harzianum*. J. King Saud Univ. Sci..

[384] Al-Hazmi A.S., TariqJaveed M. (2016). Effects of different inoculum densities of *Trichoderma harzianum* and *Trichoderma viride* against *Meloidogyne javanica* on tomato. Saudi J. Biol. Sci..

[385] Malolepsza U., Nawrocka J., Szczech M. (2017). *Trichoderma virens* 106 inoculation stimulates defense enzyme activities and enhances phenolic levels in tomato plants leading to lowered *Rhizoctonia solani* infection. Biocontrol Sci. Technol..

[386] Yan Y., Mao Q., Wang Y., Zhao J., Fun Y., Yang Z., Peng X., Zhang M., Bai B., Liu A. (2021). *Trichoderma harzianum* induces resistance to root-knot nematodes by increasing secondary metabolite synthesis and defense-related enzyme activity in *Solanum lycopersicum* L.. Biol. Control.

[387] Islam M.M., Hossain D.M., Nonaka M., Harada N. (2017). Biological control of tomato collar rot induced by *Sclerotium rolfsii* using *Trichoderma* species isolated in Bangladesh. Arch. Phytopathol. Plant Prot..

[388] Rubio M.B., Dominguez S., Monte E., Hermosa R. (2012). Comparative study of *Trichoderma* gene expression in interactions with tomato plants using high density oligonucleotide microarrays. Microbiology.

[389] Sani M.N.H., Hasan M., Uddain J., Subramaniam S. (2020). Impact of application of *Trichoderma* and biochar on growth, productivity and nutritional quality of tomato under reduced N-P-K fertilization. Annal Agric. Sci..

[390] Natsiopoulos D., Tziolias A., Lagogiannis I., Mantzoukas S., Eliopoulos P.A. (2022). Growth-promoting and protective effect of *Trichoderma atrobrunneum* and *T. simmonssi* on tomato against soil-borne fungal pathogens. Crops.

[391] Tripathi R., Keswani C., Tewari R. (2021). *Trichoderma koningii* enhances tolerance against thermal stress by regulating ROS metabolism in tomato (*Solanum lycopersicum* L.) plants. J. Plant Interact..

[392] Mona S.A., Hashem A., Abd Allah E.F., Alqarawi A.A., Soliman D.W.K., Wirth S., Egamberdieva D. (2017). Increased resistance of drought by *Trichoderma harzianum* fungal treatment correlates with increased secondary metabolites and proline content. J. Integr. Agric..

[393] Shanmugaraj C., Kamil D., Kundu A., Singh P.K., Das A., Hussain Z., Gogoi R., Shashnak P.R., Gangaraj R., Chaithra M. (2023). Exploring the potential biocontrol isolates of *Trichoderma asperellum* for management of collar rot disease in tomato. Horticulturae.

[394] Sharma A., Salwan R., Sharma V. (2022). Extracellular proteins of *Trichoderma* and their roles in plant health. S. Afr. J. Bot..

[395] Vinale F., Sivasithamparam K., Ghisalberti E.L., Marra R., Barbetti M.J., Li H., Woo S.L., Lorito M. (2008). A novel role for *Trichoderma* secondary metabolites in the interactions with plants. Physiol. Mol. Plant Pathol..

[396] Carillo P., Woo S.L., Comite E., El-nakhel C., Rouphael Y., Fusco G.M., Borzacchiello A., Lanzuise S., Vinale F. (2020). Application of *Trichoderma harzianum*, 6-Pentyl-a-Pyrone and plant biopolymer formulations modulate plant metabolism and fruit quality of plum tomatoes. Plants.

[397] De Palma M., Docimo T., Guida G., Salzano M., Albrizio R., Giorio P., Ruocco M., Tucci M. (2021). Transcriptome modulation by the beneficial fungus *Trichoderma longibrachiatum* drives water stress response and recovery in tomato. Environ. Experim. Bot..

[398] Andrzejak R., Janowska B., Renska B., Kosiada T. (2021). Effect of *Trichoderma* spp. and fertilization on the flowering of *Begonia* X *tuberhybrida* voss. Picotee sunburst. Agronomy.

[399] Ghosh S.K., Pal S., Banerjee S. (2022). Identification and pathogenicity *Alternaria alternata* causing leaf blight of *Bacopa monnieri* (L.) Wettst. and its biocontrol by *Trichoderma* species in agrifields—An ecofriendly approach. J. Appl. Res. Med. Aromat Plants.

[400] Villa-Rodriguez E., Lugo-Enriquez C., Ferguson S., Parra-Cota F.I., Cira-Chavez L.A., Santos-Villalobos S.D.I. (2022). *Trichoderma harzianum sensu lato* TSM39: A wheat microbiome fungus that mitigates spot blotch disease of wheat (*Triticum turgidum* L. subsp. *durum*) caused by *Bipolaris sorokiniana*. Biol. Control.

[401] Javaid A., Sli S. (2011). Herbicidal activity of culture filtrates of *Trichoderma* spp. against two problematic weeds of wheat. Nat. Prod. Res..

[402] Makhlouf K.E., Boungab K., Mokrani S. (2023). Synergistic effect of *Pseudomonas azotoformans* and *Trichoderma gamsii* in management of *Fusarium* crown rot of wheat. Arch. Phytopathol. Plant Prot..

[403] Alvarez-Garcia S., Manga-Robles A., Encina A., Gutierrez S., Casquero P.A. (2022). Novel culture chamber to evaluate in vitro plant-microbe volatile interactions; Effects of *Trichoderma harzianum* volatiles on wheat plantlets. Plant Sci..

[404] Yu C., Dou K., Wang S., Wu Q., Ni M., Zhang T., Lu Z., Tang J., Chen J. (2020). Elicitor hydrophobin Hyd1 interacts with ubiquilin1-like to induce maize systemic resistance. J. Integr. Plant Biol..

[405] Karuppiah V., He A., Lu Z., Wang X., Li Y., Chen J. (2022). *Trichoderma asperellum* GDFS1009-mediated maize resistance against *Fusarium graminearum* stalk rot and mycotoxin degradation. Biol. Control.

[406] Abbey J.A., Percival D., Anku K., Schilder A., Asiedu S. (2022). Managing *Botrytis* blossom bligh of wild blueberry through field sanitation, lime sulfur and *Trichoderma* application. Can. J. Plant Pathol..

[407] Bello F., Montironi I.D., Medina M.B., Munitz M.S., Ferreira F.V., Williman C., Vazquez D., Cariddi L.N., Musumeci M.A. (2022). Mycofumigation of postharvest blueberries with volatile compounds from *Trichoderma atroviride* IC-11 is a promising tool to control rots caused by *Botrytis cinerea*. Food Microbiol..

